# Altered Neuromuscular Time–Frequency Organization During Sit-to-Walk in Parkinson’s Disease: An Explainable Artificial Intelligence Approach Using Surface Electromyography

**DOI:** 10.3390/s26185909

**Published:** 2026-09-18

**Authors:** Hwayoung Park, Changhong Youm, Sang-Myung Cheon, Bohyun Kim, Juseon Hwang, Minsoo Kim

**Affiliations:** 1 Biomechanics Laboratory, Dong-A University, Saha-gu, Busan 49315, Republic of Korea; app00113@donga.ac.kr (H.P.); 2177638@donga.ac.kr (B.K.); 2577561@donga.ac.kr (J.H.); 2677610@donga.ac.kr (M.K.); 2 Department of Health Sciences, Graduate School, Dong-A University, Saha-gu, Busan 49315, Republic of Korea; 3 Department of Neurology, School of Medicine, Dong-A University, Seo-gu, Busan 49315, Republic of Korea

**Keywords:** Parkinson’s disease, surface electromyography, sit-to-walk, machine learning, convolutional neural networks, explainable artificial intelligence

## Abstract

**Highlights:**

**What are the main findings?**

- Motor dysfunction during the sit-to-walk (STW) task in individuals with Parkinson’s disease (PD) is primarily caused by distorted neuromuscular timing and intermuscular coordination, rather than simply reduced muscle activation.
- Impaired predictive control and maladaptive proximal muscle coordination during STW phase 1 represent core features of PD.

**What are the implications of the main findings?**

- Surface electromyography analysis may provide a meaningful framework for exploring PD motor pathophysiology and developing rehabilitation strategies that improve transition movement timing and intermuscular coordination.
- The findings advance existing models of PD motor dysfunction and provide a physiological foundation for interpreting explainable machine learning-based digital neuromuscular biomarkers.

**Abstract:**

Neuromuscular dysfunction during the sit-to-walk (STW) task in Parkinson’s disease (PD) remains poorly understood. We aimed to analyze surface electromyography (sEMG) signals across STW phases using explainable machine learning (ML) and deep learning approaches in PD. Individuals with PD (*n* = 101) and healthy controls (*n* = 50) performed a standardized STW task while sEMG signals were recorded bilaterally from eight lower-limb muscles. These signals were preprocessed and segmented into three phases in the STW task. Feature-based ML models were compared with convolutional neural networks (CNN) trained on wavelet-transformed sEMG spectrograms. Explainable artificial intelligence methods identified physiologically interpretable neuromuscular patterns. STW phase 1 was the most sensitive interval for detecting PD-related neuromuscular abnormalities. Among the feature-based classifiers, the random forest model demonstrated the highest performance under five-fold cross-validation, with an accuracy of 77.5% and an area under the receiver operating characteristic curveof 0.795. SHapley Additive exPlanations analysis identified mean and median frequency during STW phase 1, left biceps femoris integrated sEMG amplitude, and left rectus femoris–biceps femoris short head co-contraction as major contributors to classification. CNN-based analyses revealed earlier and temporally concentrated activation patterns in individuals with PD, particularly in proximal muscles critical for momentum generation and postural stabilization. Overall, individuals with PD exhibited distinct temporal, spectral, and coordinative patterns of lower-limb neuromuscular activity during the STW transition. The findings support the potential of sEMG-based explainable AI for characterizing PD-associated neuromuscular patterns during functional transitions.

## 1. Introduction

Parkinson’s disease (PD) is a progressive neurodegenerative disease characterized by bradykinesia, rigidity, tremors, postural instability, and gait dysfunction [1,2,3,4]. Beyond the reduced amplitude or velocity of movement observed in these cardinal motor symptoms, PD is increasingly understood as a neuromuscular control disorder, particularly associated with deficits in the anticipatory and feedforward mechanisms essential for movement initiation and functional transitions. Deficits in the timing and coordination of muscle activation play a key role here. These deficits lead to abnormal gait and balance control, increased risk of falls, and persisting symptoms despite optimal pharmacological or surgical treatment, which leads to progressive functional decline [5]. Therefore, objective biomarkers derived from dynamic functional tasks that explicitly assess anticipatory postural control and intermuscular coordination have become crucial for elucidating the neuromuscular mechanisms underlying PD-related motor dysfunction and for more precisely characterizing disease-specific control deficits [6,7].

Clinical assessments such as the Unified Parkinson’s Disease Rating Scale (UPDRS) and the Hoehn and Yahr (H&Y) scale remain the gold standard for evaluating motor symptoms in individuals with PD [8,9]. However, these instruments rely primarily on clinician observation, are susceptible to inter-rater variability, and provide limited temporal resolution for capturing subtle, phase-specific abnormalities in neuromuscular control—particularly during movement initiation and transitional tasks [9,10]. Despite extensive research efforts, no definitive biomarkers for PD have yet been established [5], and conventional clinical scales often fail to reflect the complexity and heterogeneity of neuromuscular dysfunction that underlies functional motor impairment [1,11]. These limitations underscore the need for objective, high-resolution measures capable of quantifying task- and phase-specific motor control deficits in PD.

Advances in wearable sensing and physiological signal processing technology have made it possible to analyze movement-related neuromuscular activity at high resolution. Particularly, surface electromyography (sEMG) offers a noninvasive method for capturing muscle activation timing, intermuscular coordination, and co-contraction with high temporal resolution and is widely used in studies of gait and postural control. In this context, the sit-to-walk (STW) task should not be viewed simply as a challenging functional activity, but rather as a pathophysiological stress test that places significant demands on neuromuscular control. This is because the STW task simultaneously demands anticipatory postural adjustments (APA), proximal joint stabilization, momentum generation, and gait initiation within a narrow time window, making it particularly sensitive to deficits in feedforward control and intermuscular coordination. Existing sEMG studies of the STW task have primarily focused on the distal ankle muscles, demonstrating early activation of the tibialis anterior (TA) during momentum transfer, followed by activation of the quadriceps femoris and hamstrings during the extension and stabilization phases [12]. In individuals with PD, altered lower-limb activation patterns—such as excessive TA activity, reduced gastrocnemius activation, and abnormal muscle timing—have been described and commonly interpreted as manifestations of impaired neural control or compensatory stabilization strategies [13,14]. However, the majority of studies have focused on distal joint control, steady-state gait, or individual movement phases, and have primarily relied on average time-domain features and conventional linear analyses. Consequently, the extent to which PD-related neuromuscular dysfunction during the STW task reflects phase-specific timing and coordination deficits—particularly involving proximal hip–knee muscle coordination critical for anticipatory postural control and gait initiation—remains insufficiently understood.

Machine learning (ML) and deep learning (DL) approaches have been widely applied to sEMG and gait data to classify individuals with PD and healthy individuals and identify discriminative motor features of PD. Although these studies have confirmed well-known phenomena in PD, such as increased muscle co-contraction and abnormal timing, their contribution to pathophysiological understanding has been limited. In particular, convolutional neural networks (CNN) trained with time–frequency representations—such as wavelet-based spectrograms—offer a way to overcome these limitations by preserving the temporal organization of neuromuscular signals and capturing complex, phase-dependent patterns that are difficult to quantify using manually extracted features alone [15,16,17]. Importantly, the value of CNN in PD lies not in improving classification accuracy but in elucidating when and where neuromuscular control deteriorates during functionally demanding transitions. Explainable artificial intelligence (XAI) further extends these capabilities by transforming DL models into hypothesis-generating tools. Techniques such as gradient-weighted class activation mapping (Grad-CAM) visualize temporally and spatially distinct activation patterns, allowing for the exploration of muscle and phase-specific timing and coordination abnormalities. When applied to the STW task, the explainable CNN framework acts as a pathophysiological microscope, shifting the focus from reduced activation magnitude—a key feature of PD-related motor dysfunction—to timing and coordination distortions.

From this perspective, in this study, we aimed to apply an explainable DL framework to sEMG signals from proximal and distal lower limb muscles to analyze neuromuscular control during the STW task in individuals with PD. The primary objective of this study was not simply to improve classification performance or diagnostic sensitivity but to elucidate how phase-specific neuromuscular timing, intermuscular coordination, and proximal control strategies are reorganized during the functionally demanding transitions task in individuals with PD. To this end, we integrated wavelet-based time–frequency representations with CNN-based modeling and XAI techniques to enable mechanistic analysis of sEMG signals. Within this framework, the identification of explainable sEMG-based biomarkers is considered a secondary outcome, aimed at supporting and complementing interpretations based on physiological context. Therefore, by reinterpreting the STW task as a tool for exploring neuromuscular control, we aimed to go beyond simple observations of activation magnitude and derive interpretable insights into PD-related motor dysfunction.

## 2. Materials and Methods

### 2.1. Participants

We recruited 101 individuals with PD (aged 44–83 years) and 50 healthy controls (HCs) (aged 53–77 years). Age was included as a covariate in subsequent analyses to control for potential age effects. PD diagnosis was confirmed by a neurologist based on the Movement Disorder Society Clinical Diagnostic Criteria for Parkinson’s Disease [17], and UPDRS [9,18], which comprehensively assess motor and non-motor symptoms using both clinician-rated and patient-reported measures. The inclusion criteria were mild-to-moderate idiopathic PD, stable anti-Parkinsonian medication, and the ability to independently perform walking and standing tasks. The exclusion criteria included other neurological disorders, orthopedic conditions, psychiatric illnesses, significant freezing of gait, severe balance impairment, conditions compromising safe mobility, severe dysautonomia with hypotension, major depression, dementia, pregnancy, cardiac pacemaker implantation, deep brain stimulation, severe visual impairment, or vestibular dysfunction. The PD group had a mean age of 68 years, with 52.9% female participants. Approximately 49.0% of participants were classified as H&Y stage 2 [19,20], with a mean disease duration of 5.7 years and an average UPDRS Part III motor score of 29.5. Individuals with PD were significantly older than HCs and showed a small but statistically significant difference in MoCA-K scores, with the HC group showing a slightly lower mean score (Table 1). This direction was verified against the source data and was not attributable to a transcription or data-entry error. Because the groups were not prospectively matched on cognitive performance, this modest difference may reflect sampling variability or characteristics of the recruited cohort and should not be interpreted as indicating better cognitive function in individuals with PD. Age, sex, and MoCA-K were therefore included as covariates in the adjusted between-group analyses.

This study was conducted in accordance with the ethical principles of the Declaration of Helsinki and was approved by the Institutional Review Board of Dong-A University Medical Center (approval number: DAUHIRB-22-089). Participant recruitment and data collection commenced after ethical approval. All participants provided written informed consent after receiving a detailed explanation of the study objectives and procedures. The study adhered to all relevant ethical guidelines and regulations and was retrospectively registered with the Clinical Research Information Service of the Republic of Korea (KCT0009353; 19 April 2024; http://cris.nih.go.kr), after completion of participant recruitment and data collection.

### 2.2. Experimental Procedures

All participants underwent demographic and clinical assessments and performed a standardized STW task while three-dimensional kinematic and sEMG data were recorded concurrently using a motion capture system (Vicon Motion Systems Ltd., Oxford, UK) and wireless sEMG sensors (Trigno Wireless EMG System; Delsys Inc., Natick, MA, USA). Individuals with PD were evaluated in the “on” medication state, approximately 2–3 h after taking their prescribed medication.

**Figure 1:**
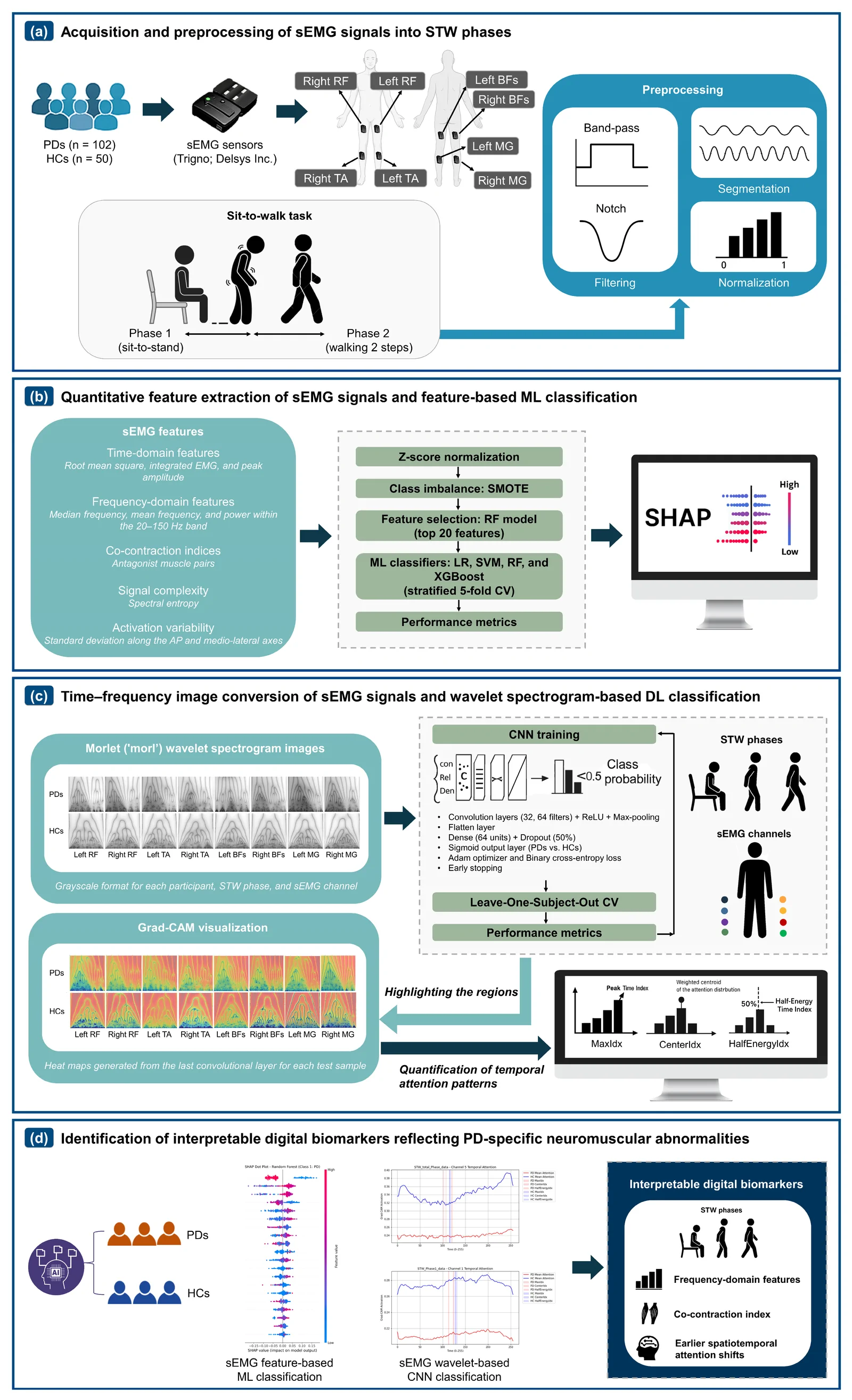
Overview of the study workflow from data acquisition to analysis. The diagram illustrates the workflow from data acquisition through feature extraction, ML and DL analysis pipelines, and explainable artificial intelligence visualization. Abbreviations: sEMG, surface electromyography; STW, sit-to-walk; PDs, individuals with Parkinson’s disease; HCs, healthy controls; RF, rectus femoris; TA, tibialis anterior; BFs, biceps femoris short head; MG, medial gastrocnemius; ML, machine learning; AP, anterior–posterior; SMOTE, synthetic minority oversampling technique; RF model, random forest model; LR, logistic regression; SVM, support vector machine with linear kernel;XGBoost, eXtreme Gradient Boosting; CV, cross-validation; SHAP, SHapley Additive exPlanations; DL, deep learning; ReLu, rectified linear unit activation; LOSO, leave-one-subject out; Grad-CAM, gradient-weighted class activation mapping; CNN, convolutional neural network; MaxIdx, maximum activation index; CenterIdx, center of mass index; HalfEnergyIdx, half-energy cumulative index.

Three-dimensional kinematic data were recorded at 100 Hz using nine infrared cameras (Vicon MX-T10, Vicon Motion Systems Ltd., Oxford, UK) positioned around the measurement space. A full-body biomechanical model was constructed using the Plug-in Gait model based on the modified Helen Hayes marker set, with 39 spherical reflective markers (14 mm in diameter) placed at standardized anatomical landmarks. In particular, trajectories of the marker placed over the seventh cervical vertebra (C7) and the bilateral heel markers were used to identify the STW phases described below. Marker placement was performed consistently by the same trained examiner across participants. The global coordinate system for the STW task was defined with the left edge of the walkway set as the origin (0, 0, 0). The *X*-axis represented the participant’s mediolateral direction, the *Y*-axis corresponded to the anterior–posterior direction of motion, and the *Z*-axis indicated the vertical direction. The experimental protocol began with the placement of dry sEMG electrodes on the skin using double-sided adhesive tape. Each sensor measured 27 × 37 × 13 mm, weighed 14 g, had a battery life of approximately 8 h, a differential input range of 11 mV/22 mV, a resolution of 16 bits, baseline noise of 0.75 µV, a maximum sampling rate of 4370 Hz, and a synchronization accuracy of <1 sampling period. Sensors were positioned on the following muscles: channel (Ch) 1, left rectus femoris (RF); Ch 2, right RF; Ch 3, left TA; Ch 4, right TA; Ch 5, left biceps femoris short head (BFs); Ch 6, right BFs; Ch 7, left medial gastrocnemius (MG); and Ch 8, right MG. These muscles were selected because they represent the primary lower-limb muscle groups involved in hip, knee, and ankle flexion–extension, which are fundamental to the STW task. Muscle locations are illustrated in Figure 1. Before electrode placement, each participant’s skin was cleaned with an alcohol swab to reduce impedance and improve electrode adherence. When necessary, hair was shaved, with the participant’s consent, to maximize electrode–skin contact. Electrodes were aligned with the muscle fibers and positioned at the midpoints of the target muscles to optimize signal quality, and the placement sites were marked to ensure consistency across trials. After setup, participants walked a short distance to verify signal quality and stability. sEMG signals were recorded at a sampling rate of 2000 Hz, following the standardized recommendations of the Surface Electromyography for Non-Invasive Assessment of Muscles guidelines.

STW data were collected while participants performed a revised timed-up-and-go task at their preferred walking speed. Each participant was instructed to stand up from an armless chair, walk to a cone placed 3 m away, circle it, and then exit the walkway. The task was repeated 6 times, with a 1-min rest interval between trials.

### 2.3. Data Analysis

A comprehensive flowchart illustrating the workflow from data acquisition to modeling and visualization is presented in Figure 1. The analysis consisted of four key steps: (1) preprocessing and segmenting sEMG signals into distinct STW phases; (2) comparing feature-based ML models with a CNN trained on wavelet-transformed spectrograms; (3) applying Grad-CAM to visualize spatiotemporal model attention across muscles and STW phases; and (4) identifying interpretable digital biomarkers that reflect PD-specific neuromuscular abnormalities.

### 2.4. STW Phase Definition and sEMG Signal Preprocessing

Motion capture and sEMG signals were recorded concurrently during each STW trial. The two data streams were temporally aligned using the common acquisition time base, and marker-derived event times were mapped to the corresponding sEMG time points for phase-specific signal extraction. Thus, the motion capture data were used to define the temporal boundaries of the STW phases, whereas the subsequent neuromuscular analyses were performed on the corresponding sEMG signals.

sEMG data were analyzed separately for the following STW phases: (1) overall phase, representing the entire STW sequence; (2) phase 1, corresponding to the sit-to-stand transition; and (3) phase 2, representing the initial walking step (Figure 1a). Phase 1 was defined as the interval beginning with the forward displacement of the seventh cervical vertebra marker and continuing through the seat-off event (pelvis lift-off from the chair) until the heel contact of the leading leg initiating the first step. Phase 2 was defined as the interval from this initial heel contact to the subsequent heel contact of the contralateral leg, thereby capturing the first complete contralateral step. During preprocessing, consistency across participants was ensured by normalizing STW trials to the right leg for the first step. The more affected side in individuals with PD and the dominant side in HCs were explicitly included as separate features to account. Raw sEMG signals sampled at 2000 Hz were processed using a fourth-order Butterworth band-pass filter (40–400 Hz) and a second-order notch filter at 60 Hz to remove movement artifacts and electrical noise. The filtered signals were then normalized using Z-scores to standardize amplitude variations across participants and sessions.

### 2.5. Quantitative Feature Extraction and Time–Frequency Image Conversion of sEMG Signals

For the feature-based analysis, sEMG features were calculated separately for each valid repetition and STW phase. Trial-level values were averaged across the six repetitions to obtain a single participant-level value for each sEMG-derived feature. For each sEMG channel and STW phase, time-domain features were calculated, including root mean square (RMS), integrated EMG (iEMG), and peak amplitude. Co-contraction indices were derived from the product of normalized signals of antagonist muscle pairs (e.g., left/right RF with left/right BFs, and left/right TA with left/right MG). Frequency-domain features, such as median frequency, mean frequency, and power within the 40–150-Hz band, were extracted using fast Fourier transform. Signal complexity was quantified using spectral entropy, with higher values indicating greater irregularity and complexity of muscle activation patterns. To assess activation variability, standard deviations (SD) were calculated along the anteroposterior (AP) and mediolateral axes. AP variability was defined as the SD of filtered sEMG signals along the *X*-axis, whereas mediolateral variability was defined as the SD of signals along the *Y*-axis, reflecting neuromuscular control in these respective planes. Detailed definitions and computational formulas for all features are provided in Supplementary Materials, Table S1. All variables were programmatically extracted using custom MATLAB scripts (MATLAB R2022b; MathWorks, Natick, MA, USA) and stored in structured Excel spreadsheets for each participant and phase to facilitate group-level statistical analysis and subsequent ML applications.

To explore phase- and muscle-specific patterns in individuals with PD and HCs, time-series sEMG signals from all eight channels were transformed into grayscale spectrogram images using continuous wavelet transform. Preprocessed sEMG signals were rectified and smoothed using an RMS envelope with a 100-ms sliding window. The Morlet (“morl”) wavelet was applied across scales corresponding to frequency ranges relevant to muscle activity. The resulting time–frequency power spectrograms were log-scaled, normalized, and resized to 256 × 256 pixels. All spectrograms were stored in grayscale format for each participant, task phase, and sEMG channel and subsequently used as inputs for CNN-based classification models to evaluate performance.

### 2.6. ML Classification and Explainable Feature Importance Analysis of sEMG Features

Feature-based ML analyses were conducted using the 66 sEMG-derived features obtained during the STW task. Classification models included logistic regression (LR), support vector machine (SVM), random forest implemented using scikit-learn (version 1.2.2),and extreme gradient boosting (XGBoost) implemented using the XGBoost package (version 2.1.2). To minimize information leakage between model development and evaluation, a stratified five-fold cross-validation (CV) procedure was implemented with feature selection per-formed independently within each training fold. For each fold, the data were first separated into training and held-out test subsets. No separate validation set was used because model hyperparameters were fixed and no validation-based hyperparameter optimization was performed. Feature standardization was performed using parameters estimated exclusively from the training subset and subsequently applied to the corresponding test subset. A random forest classifier was then fitted using only the training observations to rank the 66 candidate features according to feature importance, and the 20 highest-ranked features were retained for that fold. Class imbalance was addressed using class weighting during classifier training.

Each classifier was trained using only the selected features from the corresponding training fold and evaluated on the untouched held-out participants. Classification performance was quantified using accuracy, precision, sensitivity, specificity, F1-score, and area under the receiver operating characteristic (ROC) curve (AUC). Fold-level performance was summarized as the mean ± standard deviation across the five folds. In addition, out-of-fold (OOF) predictions from all held-out participants were pooled to construct the overall confusion matrix and ROC curve, such that each participant contributed to model evaluation exactly once. The classification pipeline was implemented in Python (version 3.11) using the scikit-learn (version 1.2.2) and imbalanced-learn (version 0.11.0) libraries.

To complement model based feature ranking, SHapley Additive exPlanations (SHAP) analysis was applied to interpret model predictions by quantifying and visualizing the contribution of each sEMG-derived variable. To interpret the random forest classifier while preserving separation between model training and evaluation, SHAP values were calculated in an out-of-fold manner. For each CV fold, a TreeExplainer was applied to the random forest model trained using the corresponding training subset, and SHAP values were calculated only for participants in the held-out test fold. SHAP values from the five non-overlapping test folds were subsequently pooled to obtain participant-level out-of-fold feature attribution. Global feature importance was determined using the mean absolute out-of-fold SHAP value. Feature importance was visualized using bar plots (highlighting the mean absolute SHAP values) and summary plots (illustrating the distribution and directionality of feature effects on the predicted class: individuals with PD vs. HCs).

### 2.7. DL Classification Based on Wavelet Spectrogram Images of sEMG Signals and Grad-CAM-Based Interpretability

To investigate spatiotemporal neuromuscular differences between individuals with PD and HCs, we implemented a DL pipeline using a CNN applied to time–frequency spectrograms derived from sEMG signals. For the CNN analysis, repeated trials were retained separately. Thus, up to six spectrogram images per participant were generated for each channel–phase combination. Each preprocessed sEMG signal was converted into grayscale wavelet spectrograms, as described previously, and used to train a CNN for each muscle channel and STW phase (Figure 1c). The final CNN datasets contained 849 spectrograms per channel for the overall STW phase, 841 per channel for phase 1, and 799 per channel for phase 2. The CNN architecture consisted of two convolutional layers (32 and 64 filters) with rectified linear unit activation and max-pooling, followed by a flattening layer, a fully connected dense layer (64 units), dropout (50%), and a sigmoid output layer for binary classification. The model was compiled using the Adam optimizer and binary cross-entropy loss. Training employed early stopping to prevent overfitting, with a validation split of 20%, a batch size of 16, a maximum of 50 epochs, and a patience of 10 epochs. Model generalizability across participants was evaluated using leave-one-subject-out (LOSO) CV for each sEMG channel and STW phase. In each fold, one participant was reserved as the test set, whereas the remaining participants were used for training. From the remaining participants, 20% were assigned to a subject-wise validation set for monitoring validation performance and early stopping, and the remaining participants were used for model training. All spectrograms belonging to an individual participant were assigned to only one of the training, validation, or test partitions. The held-out test participant was not involved in model fitting, validation, or model selection, and final CNN performance was derived exclusively from predictions for the held-out participants across LOSO iterations. Predictions were generated as class probabilities and converted into binary labels using a threshold of 0.5. Performance metrics, including accuracy, F1-score, and AUC, were calculated and averaged across folds. This procedure was repeated independently for each of the eight sEMG channels and for each STW phase.

Grad-CAM was applied to interpret CNN decision-making, explain model predictions, and visualize class-discriminative regions within the spectrogram images (Figure 1c). Heat maps were generated from the last convolutional layer for each test sample and resized to match the original spectrogram resolution (256 × 256) using NumPy arrays (.npy) for quantitative and visual analyses. These maps highlighted the regions contributing most strongly to classification. Phase- and channel-specific classification performances were compiled and exported for statistical comparison and interpretation. This pipeline enabled the identification of muscle- and phase-specific sEMG spectrotemporal features that differentiated individuals with PD from HCs.

For each Grad-CAM map, temporal attention patterns were quantified using three key metrics along the time axis (horizontal direction): (1) maximum attention index (MaxIdx), defined as the time point with the highest average activation; (2) center of mass index (CenterIdx), the weighted centroid of the attention distribution; and (3) half-energy cumulative index (HalfEnergyIdx), the time point at which 50% of the cumulative Grad-CAM energy was reached. These metrics were calculated for each participant, phase, and sEMG channel. Group differences in temporal attention between individuals with PD and HCs were assessed using independent two-sample *t*-tests. Visualization was performed by plotting group-level average Grad-CAM attention curves and overlaying the three indices. These results provide insight into when the CNN focused most strongly during the temporal course of the STW task and whether this focus systematically differed between groups, potentially reflecting STW phase-specific neuromuscular deficits. All DL analyses, including CNN training, evaluation, Grad-CAM generation, and visualization, were conducted using NumPy, TensorFlow, scikit-learn, and Matplotlib in Python.

### 2.8. Independent Temporal Validation of Grad-CAM Attribution

To evaluate whether the temporal localization of Grad-CAM attribution corresponded to independently measured characteristics of muscle activation, an additional validation analysis was performed using the raw sEMG signals. Grad-CAM maps were treated as class-discriminative attribution maps rather than as direct measurements of muscle activity.

The raw sEMG signals sampled at 2000 Hz were processed to obtain the linear envelope. Muscle activation onset was identified when the signal exceeded a base-line-dependent threshold defined as the baseline mean plus three standard deviations, for at least 50 ms. Onset latency was expressed relative to the beginning of the STW analysis window. In addition, peak activation time, amplitude-weighted temporal centroid, and the time at which 50% of cumulative EMG energy was reached were calculated for the overall STW phase, phase 1, and phase 2 and expressed as percentages of the corresponding analysis window. These independently derived sEMG measures were compared with the conceptually corresponding Grad-CAM indices: MaxIdx with peak activation time, CenterIdx with temporal centroid, and HalfEnergyIdx with half-energy time. Repeated trials were averaged within each participant before statistical analysis.

### 2.9. Statistical Analysis

Data normality was assessed using the Shapiro–Wilk test. Depending on the distribution, comparisons of demographic and clinical characteristics, as well as sEMG-derived variables, between individuals with PD and HCs were performed using independent *t*-tests for normally distributed data or Mann–Whitney *U* tests for non-normally distributed data (Supplementary Table S2). Continuous variables were reported as means and SD, whereas categorical variables were presented as frequencies and percentages. All analyses were conducted separately for each sEMG channel and STW phase.

Because age and MoCA-K scores differed significantly between the PD and HC groups, the primary between-group comparisons of sEMG- and Grad-CAM-derived variables were additionally evaluated using multivariable linear regression models to estimate covariate-adjusted group effects. For each dependent variable, group was entered as the primary independent variable (HC = 0, PD = 1), with age, sex, and MoCA-K score included as covariates:

$$Outcome=\beta 0+\beta 1\left(Group\right)+\beta 2\left(Age\right)+\beta 3\left(Sex\right)+\beta 4\left(MoCA-K\right)+\varepsilon$$

The coefficient for group (β1) represented the adjusted difference between individuals with PD and HCs. Adjusted group effects were reported as regression coefficients with corresponding 95% confidence intervals and *p*-values. To account for multiple comparisons across sEMG- and Grad-CAM-derived outcomes, *p*-values for the group effect were corrected using the Benjamini–Hochberg false discovery rate (FDR) procedure, with q < 0.05 considered statistically significant.

Correlation analyses were performed to explore relations between SHAP-based selected sEMG features and Grad-CAM-based temporal attention indices and clinical measures. Given the ordinal nature of several clinical variables and the non-normal distribution of some extracted features, Spearman’s rank correlation coefficients (ρ) were used for the correlation analyses, including to quantify the correspondence between Grad-CAM temporal indices and independently derived raw sEMG temporal measures. Statistical significance was defined as *p* < 0.05 for individual tests.

## 3. Results

### 3.1. Covariate-Adjusted Between-Group Comparisons

After adjustment for age, sex, and MoCA-K, three of the 66 sEMG-derived variables remained significantly different between individuals with PD and HCs after FDR correction. During STW phase 1, individuals with PD showed a lower left RF–BFs co-contraction index (adjusted β = −0.0189, 95% CI = −0.0286 to −0.0093, q = 0.010), whereas median (adjusted β = 19.26, 95% CI = 8.80–29.72, q = 0.013) and mean frequency (adjusted β = 15.69, 95% CI = 6.77–24.60, q = 0.015) were higher in individuals with PD. No other sEMG-derived variables remained significant after FDR correction. Complete covariate-adjusted results for all 66 sEMG-derived variables are provided in Supplementary Materials, Table S3.

### 3.2. sEMG Feature-Based Classification and SHAP Results

**Figure 2:**
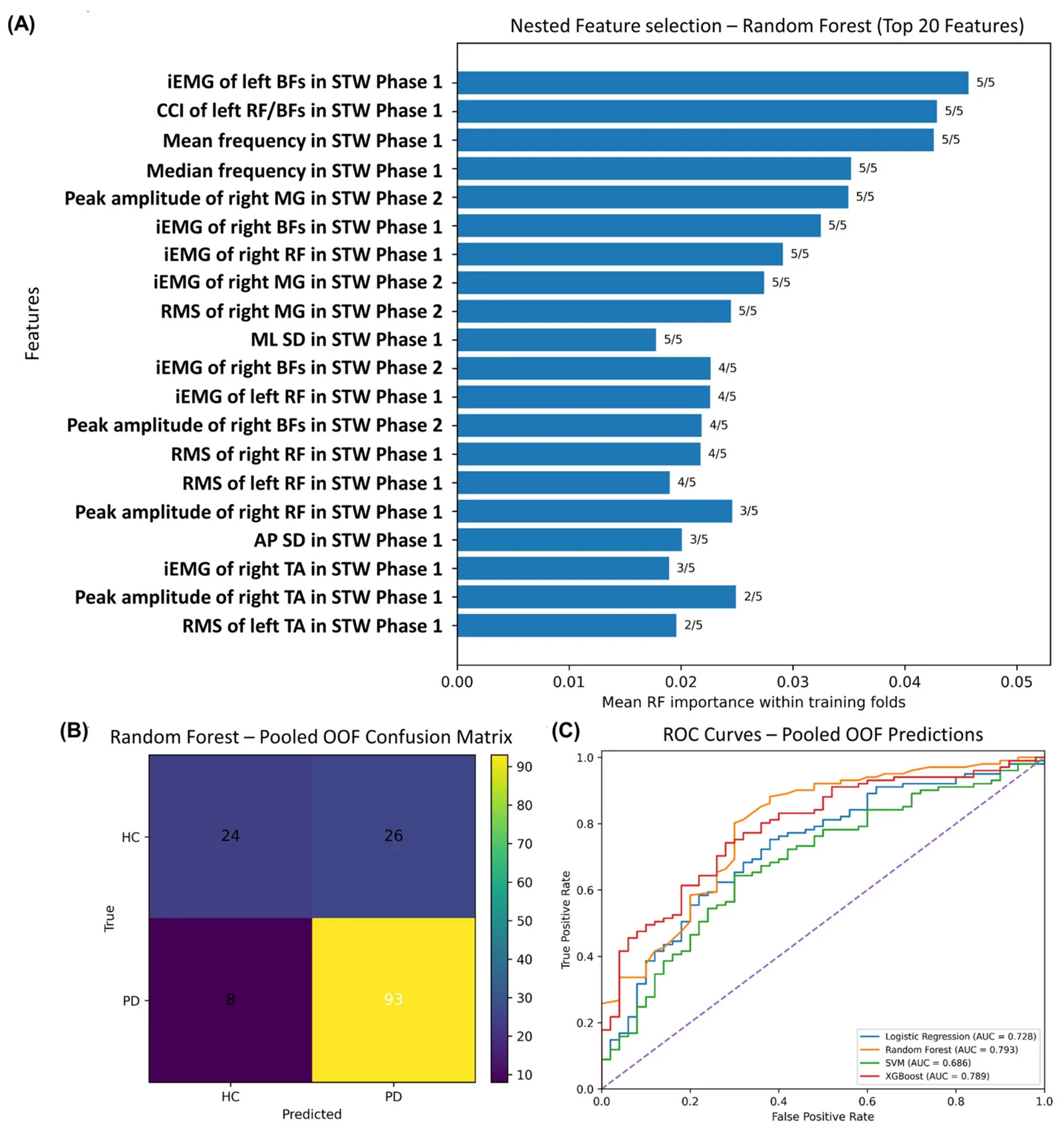
Leakage-controlled feature-based machine learning analysis for distinguishing individuals with Parkinson’s disease (PD) from healthy controls (HCs). (**A**) Stability of random forest-based feature selection across the five cross-validation training folds. Bars indicate the mean random forest importance among training folds in which each feature was selected, and labels indicate the number of folds in which the feature was retained among the top 20. (**B**) Pooled out-of-fold (OOF) confusion matrix of the random forest classifier based exclusively on the 151 original participants. (**C**) Receiver operating characteristic (ROC) curves calculated from pooled OOF probabilities for four classifiers. The dashed diagonal line represents chance-level classification performance (area under the receiver operating characteristic curve, AUC = 0.5). Abbreviations: STW, sit-to-walk; iEMG, integrated EMG; BFs, biceps femoris short head; RF, rectus femoris; MG, medial gastrocnemius; RMS, root mean square; TA, tibialis anterior; CCI, co-contraction index; AP, anteroposterior; ML, mediolateral; SD, standard deviation; ROC, receiver operating characteristic; AUC, area under the receiver operating characteristic curve; SVM, support vector machine with a linear kernel; XGBoost, extreme gradient boosting.

Following implementation of training-fold-specific feature selection, several sEMG-derived features were consistently retained across the five CV folds. Ten features were selected in all five training folds, including the iEMG amplitude of the left BFs, the left RF–BFs co-contraction index, the mean and median frequencies during phase 1, the peak amplitude of the right MG during phase 2, the iEMG amplitudes of the right BFs and RF during phase 1, the iEMG and RMS amplitudes of the right MG during phase 2, and the mediolateral SD during phase 1 (Figure 2A).

Among the four classifiers, the random forest model demonstrated the highest overall classification performance. Mean five-fold accuracy was 77.51 ± 9.75%, with a precision of 78.40 ± 7.50%, sensitivity of 92.10 ± 5.66%, specificity of 48.00 ± 19.24%, F1-score of 84.66 ± 6.55%, and AUC of 0.795 ± 0.094 (Table 2 and Figure 2C). The pooled OOF AUC was 0.793. In an age-matched sensitivity analysis comprising 49 PD–HCs pairs, the between-group age imbalance was effectively reduced (SMD = 0.042). Repeating the complete leak-age-controlled ML pipeline yielded a random forest accuracy of 73.58 ± 7.10% and a mean AUC of 0.818 ± 0.038 (pooled OOF AUC = 0.809), broadly consistent with the primary ML analysis (see Supplementary Materials, Table S4). In the pooled confusion matrix, 24 of 50 HCs and 93 of 101 participants with PD were correctly classified, whereas 26 HCs were classified as PD and 8 individuals with PD were classified as HCs (Figure 2B).

**Figure 3:**
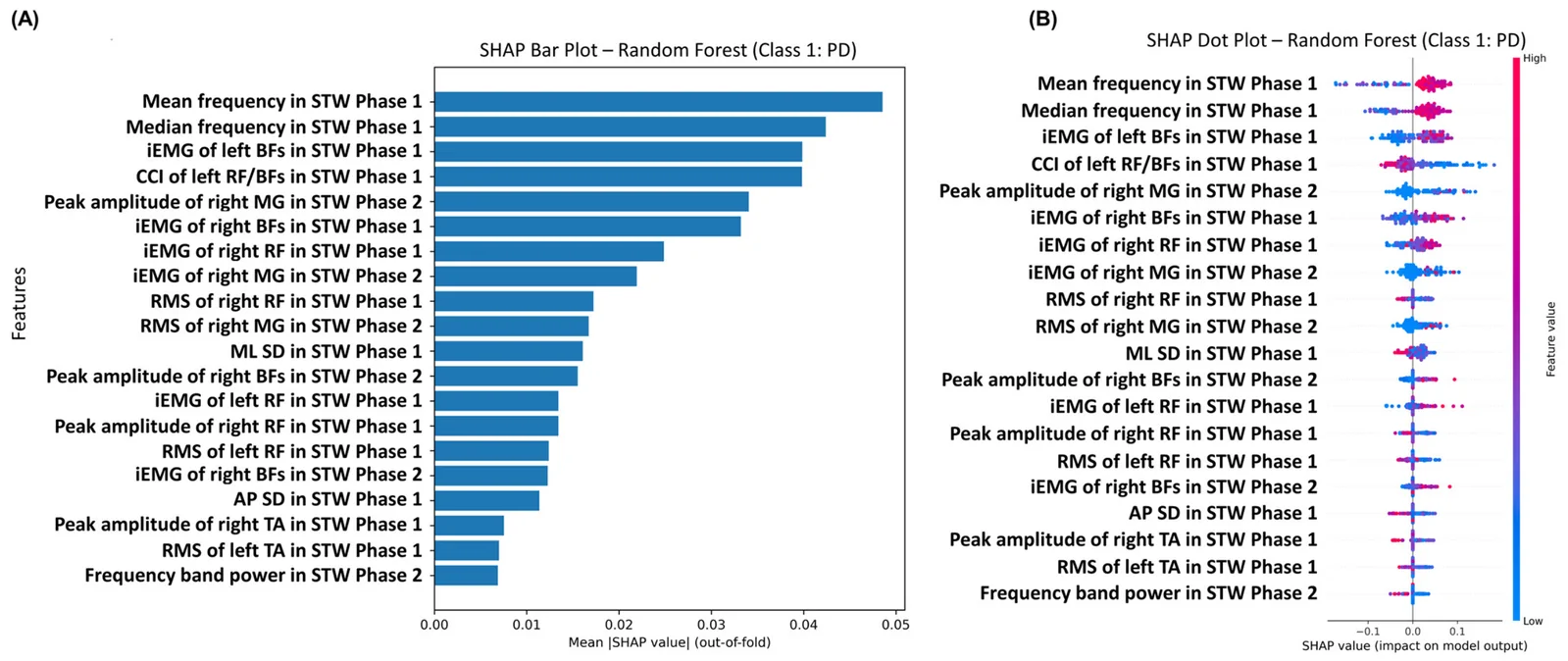
Out-of-fold (OOF) SHAP interpretation of the revised random forest classifier. (**A**) Global feature importance quantified using the mean absolute OOF SHAP value. (**B**) SHAP summary plot showing the magnitude and direction of feature contributions to classification as PD (class 1). For each cross-validation fold, SHAP values were calculated only for the held-out test participants using the corresponding fold-specific random forest model and were subsequently pooled across folds. Abbreviations: SHAP, SHapley Additive exPlanations; PD, Parkinson’s disease; STW, sit-to-walk; iEMG, integrated surface electromyography; BFs, biceps femoris short head; RF, rectus femoris; MG, medial gastrocnemius; RMS, root mean square; TA, tibialis anterior; CCI, co-contraction index; AP, anteroposterior; ML, mediolateral; SD, standard deviation.

OOF SHAP analysis provided further insights into the random forest model’s decision-making process (Figure 3). The SHAP bar plot (Figure 3A) identified the mean frequency during STW phase 1 as the most influential predictor (mean absolute SHAP value = 0.0485), followed by the median frequency (0.0424), iEMG amplitude of the left BFs (0.0399), the left RF–BFs co-contraction index during phase 1 (0.0398), and the peak amplitude of the right MG during phase 2 (0.0341). These features were consistently selected across all five training folds. The SHAP dot plot (Figure 3B) further highlighted the directionality of feature effects, showing that higher mean and median frequency values during phase 1 were associated with an increased likelihood of PD classification. Higher iEMG amplitude of the left BFs during phase 1 also tended to contribute positively to PD classification. In contrast, lower values of the left RF–BFs co-contraction index were more strongly associated with positive SHAP values, whereas higher values tended to shift the model output away from PD classification. Peak amplitude of the right MG during phase 2 also showed a meaningful contribution, with higher values generally associated with PD classification. Additional iEMG features from the right BFs and RF during phase 1 and the right MG during phase 2 further contributed to classification, indicating that the random forest model incorporated information from both frequency-domain characteristics and muscle activation magnitude and coordination.

### 3.3. CNN-Based Classification and Temporal Attention Patterns

Wavelet-transformed spectrograms of sEMG signals from the STW task were used as inputs to a CNN classifier to differentiate individuals with PD from HCs. Classification performance was evaluated separately for the overall STW phase, phase 1, and phase 2 across eight lower-limb sEMG channels (Table 3). During the overall STW phase, the right RF showed the highest accuracy (72.56%), whereas the left BFs showed the highest F1-score (82.16%) and AUC (68.79). During phase 1, the right RF achieved both the highest accuracy (69.92%) and F1-score (80.52%), while the highest AUC was observed for the left BFs (65.35). During phase 2, the right RF again showed the highest accuracy (71.59%) and F1-score (80.38%), whereas the right TA showed the highest AU C (67.42). Thus, although the right RF consistently showed the highest classification accuracy across the three STW phases, the channel associated with the highest AUC varied by phase.

Grad-CAM analysis was applied to interpret CNN decision-making by temporal attention patterns across STW phases and sEMG channels. Three quantifying indices were derived from activation maps: MaxIdx, CenterIdx, and HalfEnergyIdx. Covariate adjusted analyses of the Grad-CAM temporal indices identified several nominal between-group differences after adjustment for age, sex, and MoCA-K (Table 4). The strongest group effect was observed for the phase 2 left RF MaxIdx, which was lower in PD (adjusted β = −44.23, *p* < 0.001). Nominally lower temporal indices in PD were also observed for the phase 2 left RF HalfEnergyIdx (adjusted β = −7.43, *p* < 0.001), phase 1 left RF CenterIdx (adjusted β = −6.33, *p* < 0.001), and phase 2 left BFs HalfEnergyIdx (adjusted β = −12.40, <0.001), among other comparisons. However, none of the 72 Grad-CAM-derived group comparisons remained statistically significant after FDR correction (minimum q = 0.070). Accordingly, these temporal attention patterns were interpreted as exploratory rather than confirmatory evidence of group differences.

**Figure 4:**
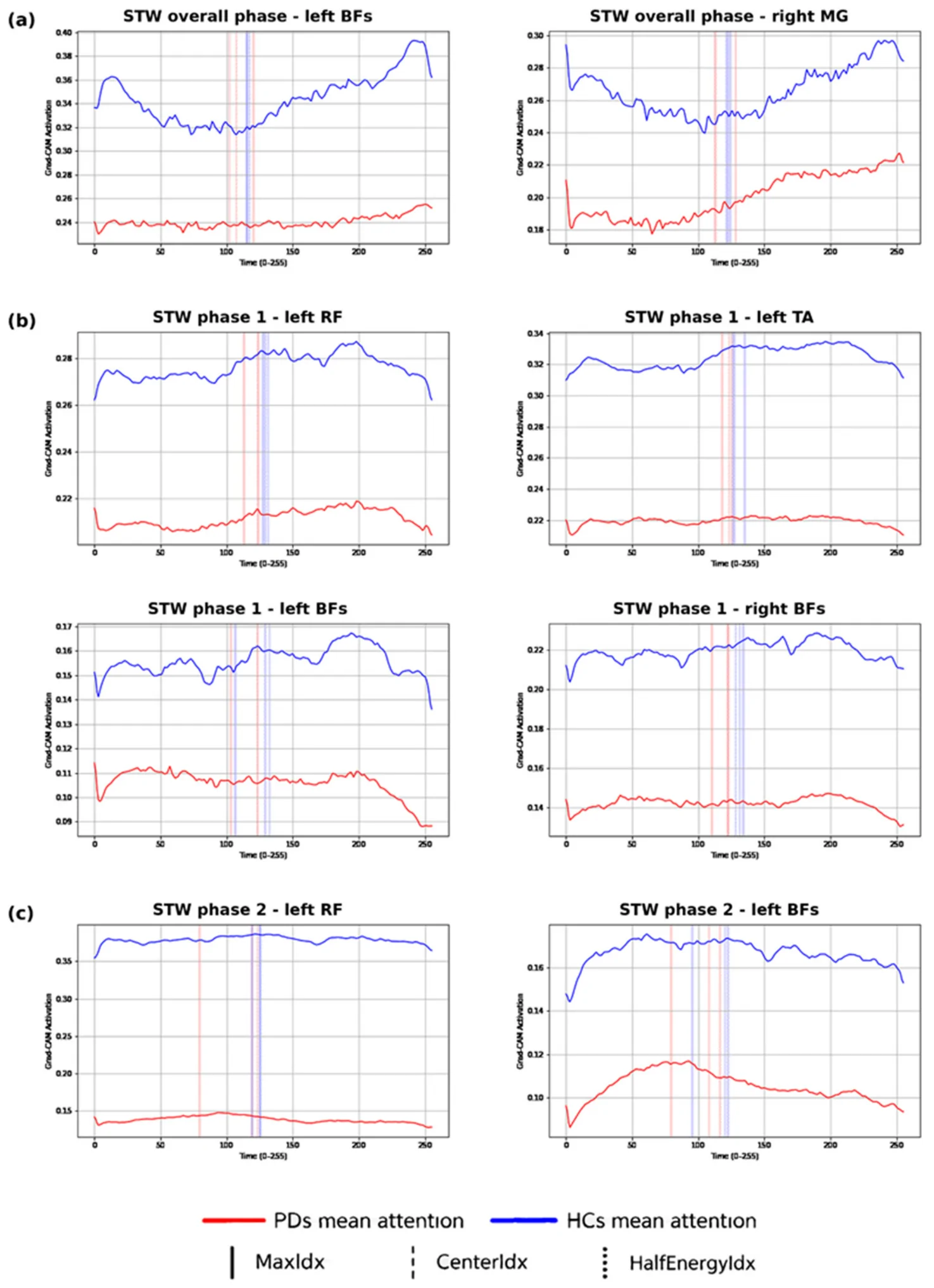
Representative temporal attention plots from CNN analysis for PD classification. Phase- and channel-specific Grad-CAM temporal attention plots highlighting regions contributing to the CNN model’s classification of individuals with Parkinson’s disease (PDs) and healthy controls (HCs). (**a**) Representative attention profiles during the overall sit-to-walk (STW) phase for the left biceps femoris short head (BFs) and right medial gastrocnemius (MG). (**b**) Representative attention profiles during STW phase 1 for the left rectus femoris (RF), left tibialis anterior (TA), left BFs, and right BFs. (**c**) Representative attention profiles during STW phase 2 for the left RF and left BFs. Red and blue curves represent the mean Grad-CAM activation profiles for PD and HC, respectively. The corresponding red- and blue-colored vertical markers indicate the temporal locations of MaxIdx, CenterIdx, and HalfEnergyIdx for each group, with the different marker styles distinguishing the three attention indices. Thus, colors consistently indicate group membership, whereas marker styles indicate the respective Grad-CAM-derived temporal indices. Abbreviations: CNN, convolutional neural network; PDs, individuals with Parkinson’s disease; HCs, healthy controls; STW, sit-to-walk; Grad-CAM, gradient-weighted class activation mapping; MaxIdx, maximum activation index; CenterIdx, center of mass index; HalfEnergyIdx, half-energy cumulative index; BFs, biceps femoris short head; MG, medial gastrocnemius; RF, rectus femoris; TA, tibialis anterior.

Representative temporal attention plots (Figure 4) revealed distinct group differences in spatiotemporal attention patterns. HCs generally demonstrated higher attention magnitudes and broader temporal distributions, whereas individuals with PD exhibited attenuated and earlier-concentrated activation. Specifically, during the overall STW phase in the left BFs (Figure 4a, left), HCs displayed a gradually increasing attention profile distributed across the task duration, whereas individuals with PD showed lower and more restricted responses. The PD group exhibited earlier and more temporally concentrated attention than HCs did, reflected by left-shifted CenterIdx and HalfEnergyIdx values and narrower attention curves. In the right MG (Figure 4a, right), individuals with PD demonstrated a steeper early rise in attention, reaching localized peaks earlier, whereas HCs exhibited broader curves characterized by gradual increases and sustained attention in later portions of the task. CenterIdx values further confirmed that individuals with PD concentrated their attention earlier than HCs did, during the overall STW phase. During STW phase 1, HCs consistently exhibited higher attention magnitudes than individuals with PD did. In the left RF, left BFs, and right BFs (Figure 4b, top left, bottom left, and right), HCs maintained higher attention throughout the phase. Temporal metrics indicated that CenterIdx and HalfEnergyIdx occurred slightly earlier in HCs, suggesting that the CNN focused on the initial portion of the phase when distinguishing groups. In the left TA (Figure 4b, top right), HCs also demonstrated higher mean attention curves, whereas individuals with PD showed earlier-shifted CenterIdx values, indicating that the model focused on TA activity earlier in individuals with PD than in HCs. During STW phase 2, HCs exhibited higher and more sustained attention in the left RF (Figure 4c, left), whereas individuals with PD showed attenuated values. In the PD group, MaxIdx occurred earlier, reflecting premature peaks relative to HCs. Similarly, HalfEnergyIdx values were shifted toward the early portion of the phase in individuals with PD, whereas in HCs they clustered around the mid-phase, reflecting gradual accumulation of attention. In the left BFs (Figure 4c, right), HCs demonstrated a rapid rise in attention at gait initiation that plateaued through mid-phase before declining. In contrast, individuals with PD showed a delayed rise, lower overall attention, and a later, reduced peak. The HalfEnergyIdx metric occurred earlier in individuals with PD, indicating that despite diminished overall attention, cumulative energy thresholds were reached more rapidly.

### 3.4. Independent Temporal Validation of Grad-CAM Attribution

Direct analysis of the sEMG signals demonstrated shorter activation onset latencies in several muscles in individuals with PD. After FDR correction, significant group differences were observed in the left RF (q = 0.030), right RF (q = 0.030), left TA (q = 0.042), and right BFs (q = 0.032). The corresponding standardized effect sizes were moderate, with Hedges’ g ranging from approximately −0.55 to −0.60 for the strongest group differences. The temporal correspondence between Grad-CAM attribution and independently derived sEMG measures varied by phase and temporal index. During phase 2, Grad-CAM CenterIdx was positively associated with the raw sEMG temporal centroid in the left RF (ρ = 0.315, q = 0.0026), left TA (ρ = 0.298, q = 0.0036), left BFs (ρ = 0.252, q = 0.0206), and left MG (ρ = 0.262, q = 0.0189). Significant associations between HalfEnergyIdx and the corresponding raw sEMG half-energy time were also observed for multiple muscles during phase 2. Conversely, correspondence between Grad-CAM MaxIdx and raw sEMG peak activation time was limited.

### 3.5. Relevance Between Key Features and Clinical Measures

**Figure 5:**
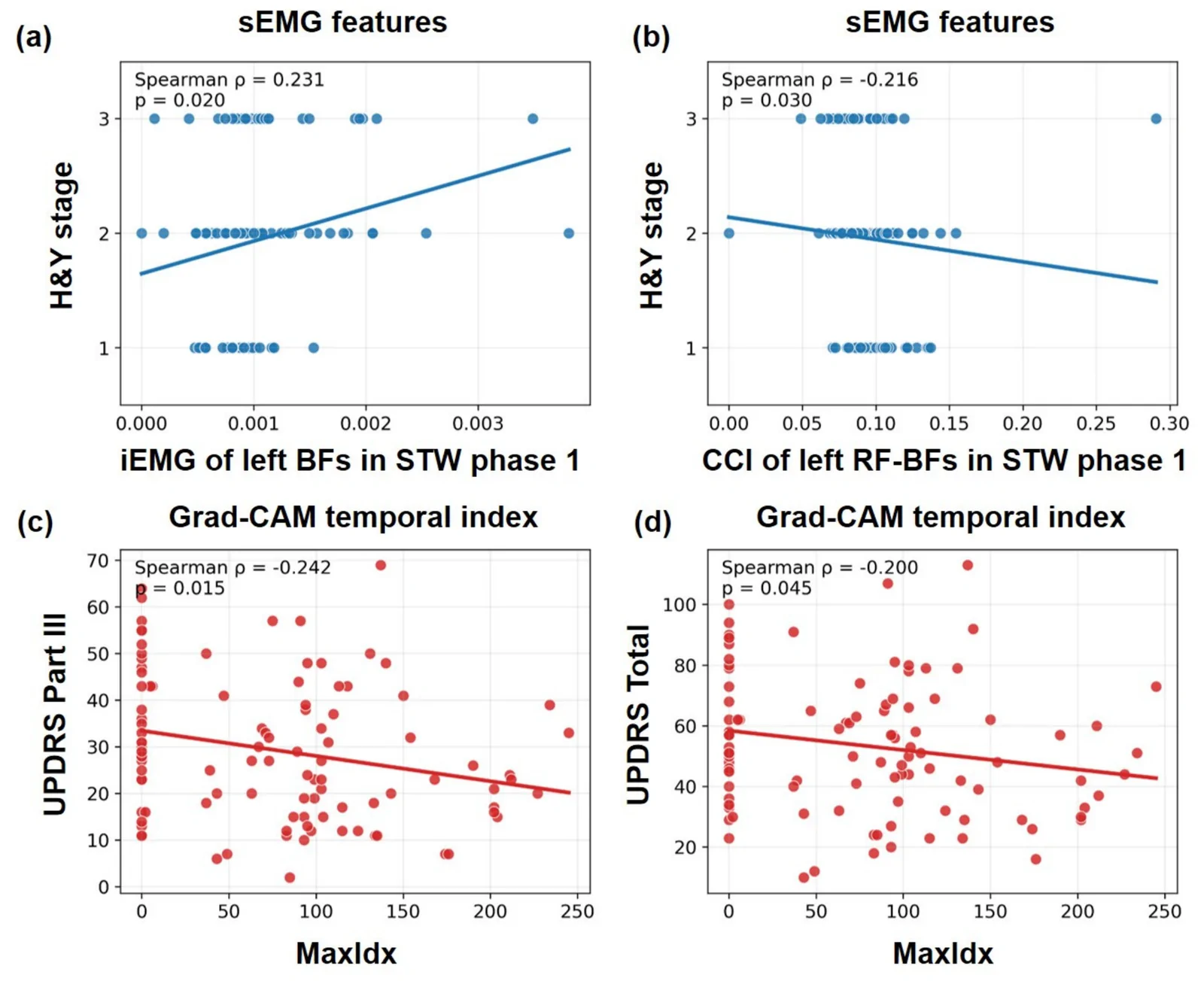
Correlations between selected sEMG features, gradient-weighted class activation mapping (Grad-CAM) temporal indices, and clinical measures of individuals with Parkinson’s disease. Scatterplots show Spearman correlations between (**a**) the iEMG amplitude of the left BFs during STW phase 1 and Hoehn and Yahr (H&Y) stages, (**b**) the co-contraction index between the left RF and left BFs in STW phase 1 and H&Y stages, and (**c**,**d**) the Grad-CAM MaxIdx of the left RF in STW phase 2 and the UPDRS Part III and Total scores. The solid lines represent the fitted trend lines. The blue dots are the sEMG features and the red dots are the Grad-CAM temporal indices. Abbreviations: sEMG, surface electromyography; Grad-CAM, gradient-weighted class activation mapping; iEMG, integrated surface electromyography; BFs, biceps femoris short head; STW, sit-to-walk; RF, rectus femoris; MaxIdx, maximum attention index; UPDRS, unified Parkinson’s disease rating scale.

To assess the clinical relevance of the sEMG features and Grad-CAM temporal indices derived from SHAP and CNN analyses for PD, we analyzed the correlations between clinical scores (Figure 5). The iEMG amplitude of the left BFs during phase 1 showed a small but significant positive correlation with the H&Y stage (Spearman’s ρ = 0.231, *p* = 0.020). Furthermore, the co-contraction index between the left RF and left BF in phase 1 was negatively correlated with the H&Y stage (ρ = −0.216, *p* = 0.030). The MaxIdx of the left RF in phase 2 showed a weak but significant negative correlation with the UPDRS Part III score (ρ = −0.242, *p* = 0.015) and UPDRS Total score (ρ = −0.200, *p* = 0.045).

## 4. Discussion

The key finding of this study is that motor dysfunction during the STW tasks in individuals with PD is primarily caused by distorted neuromuscular timing and intermuscular coordination, rather than simply reduced muscle activation [21,22,23]. A comprehensive analysis of sEMG-derived features and CNN-based temporal attention patterns revealed that muscles in PD are not simply underactivated, but rather activated in inefficient combinations at inappropriate times, particularly during the initial transitions phase of the STW tasks. These abnormalities were most pronounced during the STW phase 1, when postural control and feedforward control primarily occur [24,25,26]. A variety of analyses consistently revealed that this phase is characterized by frequency-domain shifts, excessive proximal agonist–antagonist co-contraction, and early temporal activation [23,27,28]. Importantly, this integrated pattern distinguishes our results from existing gait and EMG studies that have primarily focused on reduced activation amplitude, distal ankle muscles, or steady-state gait [29,30]. The present findings highlight the reorganization of neuromuscular control at the temporal and intermuscular coordination levels within the proximal hip–knee muscle pair during a functionally demanding transition. From this perspective, the STW task may be reinterpreted not simply as a challenging motor task but as a pathophysiological tool revealing deficits in predictive and feedforward neuromuscular control in PD [24,31]. Collectively, these findings advance existing models of PD motor dysfunction by reframing neuromuscular impairment as a question of when and how muscles are recruited and provide a physiological foundation for interpreting explainable ML-based digital neuromuscular biomarkers.

Existing studies applying ML approaches to sEMG or gait data from individuals with PD have repeatedly reported phenomena such as increased muscle co-contraction and activation timing abnormalities in PD [21,22,23,29]. However, most studies have focused on normal gait or distal ankle muscles and have interpreted these abnormalities at the level of averaged activation indices [29,30,31]. Consequently, there has been relatively limited discussion on how the observed abnormalities reorganize neuromuscular control strategies during functionally demanding transition movements. In this context, the present study broadens the scope of existing approaches by expanding the analysis beyond normal gait to the STW transition task, focusing on the proximal hip–knee muscle pairs crucial for momentum generation and postural stabilization, rather than distal muscles, and focusing on phase-specific timing and coordination structures, rather than averaged activation indices.

Within this expanded analytical framework, STW phase 1 emerged as a particularly sensitive phase for detecting PD-related neuromuscular dysfunction [23,24,25,26]. Both feature-based and CNN-based analyses consistently identified the early phase, corresponding to sit-to-stand initiation and APA, as the most useful interval for distinguishing individuals with PD from HCs. Importantly, the observed differences in STW phase 1 were not limited to activation magnitude but also involved phase-specific differences in the temporal and spectral organization of muscle activity and intermuscular coordination. The mean and median frequency during STW phase 1 were among the most stable and influential predictors in the feature-based analysis, together with integrated muscle activity and the RF–BFs co-contraction index. Rather than indicating a single underlying neurophysiological mechanism, these findings suggest that PD is associated with altered organization of neuromuscular activity during the early STW transition. Changes in or redistribution of sEMG frequency content may be related to differences in motor-unit recruitment, firing behavior, or synchronization [23,27,32]; however, these physiological mechanisms were not directly assessed in the present study. Previous studies have reported abnormalities in APA and muscle activation during gait initiation in PD, providing physiological context for the present observations [31,33]. Because STW initiation requires rapid coordination between postural preparation and movement progression, the early transition may be particularly sensitive to PD-associated alterations in neuromuscular timing and organization.

Classification performance also supports this interpretation. The leakage-controlled random forest model demonstrated moderate discriminatory performance, with a mean accuracy of 77.5% and an AUC of 0.795. Notably, the model showed high sensitivity for individuals with PD (92.1%) but relatively low specificity for HCs (48.0%), indicating that the identified neuromuscular features were more effective in detecting PD-related patterns than in reliably distinguishing HCs. This pattern may reflect the considerable variability in neuromuscular strategies and transition movements across individuals, which may blur the diagnostic boundary between PD and aging, as has been repeatedly reported in existing ML studies of neurodegenerative diseases [27,31,34]. In other words, PD-related neuromuscular dysfunction during the STW task may not manifest as uniform muscle weakness, but rather as subtle alterations in the temporal, spectral, and coordinative organization of muscle activity that challenge both clinical assessment and algorithmic classification [24,25,31]. Importantly, the present analysis excluded the laterality variable and incorporated feature selection entirely within the training folds, thereby reducing the possibility that classification performance was driven by group-specific laterality information. Even after this methodological refinement, frequency-domain features during STW phase 1 remained among the most stable and influential predictors, together with integrated sEMG amplitude and co-contraction characteristics. The OOF SHAP analysis similarly identified the mean and median frequencies, left BFs iEMG, and the left RF–BFs co-contraction index during phase 1 among the leading contributors to classification. These findings suggest that the discriminatory information captured by the feature-based model is more closely related to alterations in the temporal and spectral organization and coordination of neuromuscular activity during the early STW transition than to laterality itself [23,29,30].

SHAP analysis in this study was used not only to rank importance but also to generate mechanistic hypotheses regarding PD-related neuromuscular control [31,35]. Specifically, the prominent contribution of the RF–BFs co-contraction index during STW phase 1 should not be interpreted solely as a driver of classification; rather, it suggests that the coordination between proximal agonist–antagonist muscles contains information relevant to distinguishing individuals with PD from HCs [21,22,33]. In the OOF SHAP analysis, lower values of the left RF–BFs co-contraction index tended to contribute toward PD classification, whereas higher values generally shifted the model output away from PD. This pattern may instead reflect altered regulation or redistribution of agonist–antagonist coordination during the early STW transition. Previous studies have reported altered agonist–antagonist co-activation and abnormalities in muscle coordination during postural and balance tasks in individuals with PD [22,28,36]. Such findings provide a relevant physiological context for interpreting the importance of the RF–BFs co-contraction feature; however, the present cross-sectional analysis cannot determine whether the observed pattern reflects altered reciprocal inhibition, compensatory stabilization, disease-related changes in muscle recruitment, or other mechanisms. Similarly, although basal ganglia–cortical circuits are known to contribute to movement initiation and the regulation of coordinated motor activity [26,33], the present sEMG measurements do not directly assess these neural circuits. The importance of proximal RF–BFs coordination may therefore be considered consistent with altered neuromuscular organization during movement initiation rather than evidence of a specific basal ganglia-mediated mechanism. Furthermore, although the correlations between SHAP-ranked sEMG features and H&Y stages were generally weak, these exploratory correlations suggest that the identified neuromuscular indices are not simply classification artifacts but may have meaningful associations with PD clinical severity [31,33]. Therefore, longitudinal studies will be required to determine whether these patterns change with disease severity and whether they represent compensatory, consequential, or potentially contributory characteristics of PD-related motor impairment.

These results demonstrate that explainable ML approaches can function as hypothesis-generating tools, beyond mere post hoc explanations [31,35,37]. By linking SHAP-ranked feature importance to specific neuromuscular control strategies, this study supports a physiological hypothesis in which PD-related motor dysfunction during the STW task results from distorted timing [23], failure of reciprocal inhibition [22], and maladaptive proximal coordination [23,24,30]. Although speculative in nature, these interpretations provide testable hypotheses for future studies incorporating direct neurophysiological measurements, longitudinal designs, or experimental manipulation of movement preparation and gait initiation.

Unlike feature-based ML approaches, CNN analysis using wavelet spectrograms as input preserves the time–frequency structure inherent in sEMG signals, enabling direct exploration of the temporal organization of neuromuscular activity during the STW task [27,34]. To our knowledge, this study is particularly valuable in that it is one of several attempts to interpret how CNNs utilize information from specific muscles and time points to make discriminatory decisions using sEMG data-based Grad-CAM during the STW task in individuals with PD [37,38]. Grad-CAM analysis revealed that individuals with PD consistently exhibited earlier, temporally focused attention patterns in multiple muscles throughout the STW task. This pattern suggests maladaptive timing strategies during movement initiation and transition, rather than simple activation reductions or delays [24,28,33]. Specifically, abnormally early focused attention was observed in the RF, BFs, and TA during STW phase 1, during a period requiring APA and momentum transfer [24,25,26]. Temporal differences were also observed in the RF and BFs during STW phase 2. These findings may be compatible with altered temporal organization of neuromuscular activity during movement initiation and progression [24,25,33], but the Grad-CAM maps do not directly quantify feedforward control, propulsion generation, or specific neural motor commands. Nevertheless, the CNN-based observations complement the feature-based findings, which identified phase 1 frequency-domain characteristics and proximal RF–BFs coordination as important discriminative features, further suggesting that the early STW transition contains relevant PD-associated neuromuscular information [23,34].

Although CNN-based classification performance did not surpass that of the feature-based models, the purpose of the CNN analysis in this study was not solely to maximize classification accuracy but also to characterize the time–frequency information contributing to model discrimination. This approach is consistent with previous work showing that DL models applied to spectrogram representations can capture complex temporal and spectral patterns, although their performance may be sensitive to sample size, data heterogeneity, and task complexity, particularly in clinical populations [31,39]. Quantitative temporal indices (MaxIdx, CenterIdx, and HalfEnergyIdx) derived from Grad-CAM identified differences in the temporal distribution of class-discriminative attribution between individuals with PD and HCs, and some indices showed weak exploratory associations with UPDRS total and motor scores [31]. Importantly, Grad-CAM identifies regions of the sEMG time–frequency representation that inform CNN discrimination but does not directly measure physiological muscle activation or neural motor commands. Accordingly, the earlier Grad-CAM localization observed in individuals with PD should be interpreted as an earlier temporal concentration of class-discriminative information rather than as direct evidence of premature muscle activation [35]. To further clarify the physiological relevance of these temporal attribution patterns, an independent raw sEMG analysis was performed. Individuals with PD showed shorter activation onset latencies in the bilateral RF, left TA, and right BFs, while Grad-CAM CenterIdx and HalfEnergyIdx demonstrated modest but significant correspondence with independently derived sEMG temporal centroid and half-energy measures, particularly during STW phase 2. This correspondence was not uniform across all muscles, phases, or Grad-CAM indices, and MaxIdx showed limited agreement with conventional sEMG peak activation timing. Thus, indicating that Grad-CAM should not be regarded as a direct surrogate for physiological EMG events. Rather, the CNN and Grad-CAM analyses provide complementary information regarding class-discriminative time–frequency organization that partially overlaps with, but is not equivalent to, conventional measures of muscle activation timing [31,35].

This study has certain limitations, including the relatively limited sample size and the fact that all analyses were conducted under controlled laboratory conditions. Future studies should include a larger and more diverse cohort and further validate the clinical applicability and external validity of this approach through functional tasks performed under real-world conditions [31]. Another limitation is that the study was registered retrospectively with CRIS after completing participant recruitment and data collection. Although the study protocol received institutional ethical approval before participant recruitment and all study procedures were conducted under the approved protocol, retrospective registration limits independent verification that all reported outcomes and analyses were prospectively prespecified and therefore represents a methodological limitation. Future studies should be prospectively registered before participant enrollment. All individuals with PD were assessed in the clinically defined ON-medication state. Dopaminergic medication may modify gait initiation and neuromuscular control characteristics. Future studies incorporating both ON- and OFF-medication assessments are needed to determine the medication responsiveness of the identified neuromuscular and time–frequency patterns. This study focused exclusively on the STW task. Although the STW task is particularly sensitive for capturing predictive postural adjustments and movement initiation deficits in PD, it does not encompass the full spectrum of functionally relevant movements important in daily life, such as turning, direction changes, and steady-state walking [24,25]. Extending this framework to multiple transitional and gait tasks will be essential to establish the robustness and ecological validity of the proposed neuromuscular indices and digital biomarkers. The PD and HC groups showed modest but statistically significant differences in age and MoCA-K scores, with the HC group showing a slightly lower MoCA-K mean. This direction was verified against the source data and may reflect sampling variability or characteristics of the recruited cohort because the groups were not prospectively matched on cognitive performance. Although age, sex, and MoCA-K were included as covariates, residual demographic and cognitive confounding cannot be excluded. Future studies using age-, sex-, and cognition-matched cohorts are warranted. Although Grad-CAM was used to interpret the CNN model in this study, relying on a single XAI technique may limit the depth of mechanistic interpretation. Grad-CAM offers the advantage of intuitively presenting spatiotemporal attention domains; however, it has limitations in fully explaining the model’s decision-making process or cross-validating different interpretation perspectives [35,37]. Because the CNNs in this study were trained separately for each sEMG channel and STW phase, they could not directly model the interactions between multiple muscles or the dynamic coupling between STW phases. Therefore, future studies need to introduce a modeling framework that can comprehensively analyze multiple types of information to more precisely elucidate PD-specific neuromuscular synergy and temporal organization [30,31]. Although the independent raw sEMG analysis demonstrated partial temporal correspondence with the Grad-CAM indices, Grad-CAM remains a model-specific attribution method and should not be regarded as a direct physiological measure of muscle recruitment or neural feedforward control. The correspondence was modest and phase- and muscle-specific; notably MaxIdx in particular did not consistently correspond to conventional peak muscle activation timing. Accordingly, the Grad-CAM findings are interpreted as class-discriminative temporal–frequency attribution patterns rather than precise localization of physiological activation events. Furthermore, the clinical correlation analyses were exploratory and were not adjusted for multiple testing, meaning that the nominal associations observed with clinical severity may include false-positive findings and therefore require independent confirmation. Finally, longitudinal studies are needed to definitively assess the clinical utility of the neuromuscular timing and coordination indices proposed in this study. Future studies should follow up individuals with PD over the long term to determine how these indices relate to disease progression, fall risk, or functional improvements following rehabilitation interventions [32,40].

## 5. Conclusions

This study demonstrated distinct temporal, spectral, and coordinative characteristics of lower-limb sEMG during the STW transition in individuals with PD compared with HCs, as revealed through a dual framework integrating feature-based ML and wavelet spectrogram-based CNN modeling. Feature-based analysis identified phase-specific neuromuscular features that contributed to group discrimination, while wavelet-based CNN and explainability analyses characterized class-discriminative time–frequency patterns across the STW transition. Independent raw sEMG analysis provided complementary evidence of altered activation timing and partial correspondence with CNN-derived temporal attribution. These findings are consistent with altered neuromuscular organization during movement preparation and gait initiation in PD. Furthermore, explainable ML and DL may be considered not only as PD classification tools but also as pathophysiological hypothesis-generating tools to elucidate when and where neuromuscular control breaks down during functionally demanding movement transitions. However, because the present study was cross-sectional and observational, the identified sEMG patterns should be interpreted as PD-associated characteristics rather than causal mechanisms of motor dysfunction. Further longitudinal, interventional, and neurophysiological studies are required to determine whether these patterns represent contributors to, consequences of, or compensatory responses to PD-related motor impairment.

## Figures and Tables

**Table 1:** Demographics and clinical characteristics of the study participants.

	Individuals with PD (*n* = 101)	Healthy Controls (*n* = 50)	*p*-Value
Sex (male/female)	47/54	22/28	0.863 ^a^
Age (y)	68.10 ± 7.22	65.44 ± 5.47	**0.023 ^b^**
Height (cm)	160.32 ± 8.46	161.14 ± 8.38	0.574 ^b^
Body weight (kg)	62.89 ± 10.69	63.58 ± 10.00	0.461 ^c^
BMI (kg/m^2^)	24.38 ± 3.08	24.37 ± 2.43	0.620 ^c^
Disease duration (y)	5.61 ± 4.38	-	-
Treatment duration (y)	4.69 ± 4.27	-	-
L-dopa equivalent dose (mg/day)	537.24 ± 279.22	-	-
H&Y stage (I/II/III), n	28/50/23	-	-
UPDRS Total (score)	54.24 ± 23.12	-	-
UPDRS Part III (score)	29.36 ± 15.29	-	-
MoCA-K (score)	25.84 ± 2.99	24.86 ± 2.99	**0.025 ^c^**

Data are presented as mean ± standard deviation or n (%), as appropriate, with significant differences between groups indicated in bold (*p* < 0.05). Abbreviations: PD, Parkinson’s disease; BMI, body mass index; L-dopa, levodopa; H&Y, Hoehn and Yahr; UPDRS, unified Parkinson’s disease rating scale; MoCA-K, Korean version of the Montreal cognitive assessment. ^a^ Fisher’s exact test; ^b^ Independent samples *t*-test; ^c^ Mann–Whitney *U* test.

**Table 2:** Comparison of classifier performance in distinguishing individuals with PD from HCs using leakage-controlled five-fold cross-validation.

Model	Accuracy (%)	Precision (%)	Sensitivity (%)	F1-Score (%)	AUC
LR	67.55 ± 10.90	80.66 ± 8.51	67.29 ± 11.07	73.25 ± 10.01	0.743 ± 0.127
SVM	68.88 ± 2.84	71.10 ± 3.16	91.10 ± 9.61	79.53 ± 2.83	0.709 ± 0.092
RF	77.51 ± 9.75	78.40 ± 7.50	92.10 ± 5.66	84.66 ± 6.55	0.795 ± 0.094
XGBoost	74.15 ± 4.47	80.39 ± 3.28	81.14 ± 4.36	80.74 ± 3.51	0.785 ± 0.078

Data are presented as the mean ± standard deviation across five stratified cross-validation folds; Feature standardization and random forest-based top-20 feature selection were performed independently within each training fold. Class imbalance was addressed using class weighting during model training. All test folds contained original, non-synthetic participants only. Abbreviations: PD, Parkinson’s disease; HCs, healthy controls; LR, logistic regression; SVM, support vector machine with linear kernel; RF, random forest; XGBoost, eXtreme Gradient Boosting; AUC, area under the receiver operating characteristic curve.

**Table 3:** Classification performances of the CNN model for each STW phase and channel.

STW Phase/Channel		Left RF	Right RF	Left TA	Right TA	Left BFs	Right BFs	Left MG	Right MG
Overall phase	Accuracy (%)	71.26	**72.56**	71.97	70.32	72.44	69.26	68.55	69.73
	F1-score (%)	81.71	81.78	81.69	80.85	**82.16**	80.79	80.30	80.66
	AUC	0.680	0.638	0.635	0.636	**0.** **68** **8**	0.600	0.549	0.612
Phase 1	Accuracy (%)	67.30	**69.92**	67.78	68.61	67.90	68.73	66.94	67.30
	F1-score (%)	78.83	**80.52**	79.01	79.21	78.64	79.53	79.41	79.49
	AUC	0.617	0.588	0.613	0.609	**0.** **65** **4**	0.647	0.521	0.522
Phase 2	Accuracy (%)	69.84	**71.59**	63.45	68.09	69.59	67.08	62.70	66.08
	F1-score (%)	79.42	**80.38**	75.99	78.07	78.89	77.69	75.73	77.73
	AUC	0.674	0.670	0.553	**0.** **674**	0.663	0.639	0.547	0.531

The highest performance values for each metric are indicated in bold. Abbreviations: CNN, convolutional neural network; STW, sit-to-walk; RF, rectus femoris; TA, tibialis anterior; BFs, biceps femoris short head; MG, medial gastrocnemius; AUC, area under the receiver operating characteristic curve.

**Table 4:** Comparison of temporal Grad-CAM attention metrics across STW phases and sEMG channels.

STW Phase	Channel	MaxIdx					CenterIdx						HalfEnergyIdx			
		PDs	HCs	Adjusted β (95% CI)	*p*	FDR q	PDs	HCs	Adjusted β (95% CI)	*p*	FDR q	PDs	HCs	Adjusted β (95% CI)	*p*	FDR q
Overall phase	Left RF	101.04	119.35	−26.63 (−59.97 to 6.72)	0.12	0.35	113.49	118.17	−5.62 (−12.98 to 1.74)	0.13	0.38	108.16	115.65	−8.86 (−18.71 to 1.00)	0.08	0.29
	Right RF	120.27	125.65	−0.92 (−35.70 to 33.87)	0.96	0.97	116.69	121.65	−4.66 (−11.55 to 2.23)	0.18	0.43	111.94	120.96	−8.81 (−18.41 to 0.80)	0.07	0.29
	Left TA	133.75	147.14	−13.32 (−48.64 to 21.99)	0.46	0.72	118.38	122.45	−4.15 (−11.86 to 3.56)	0.29	0.53	114.78	121.94	−7.55 (−18.13 to 3.03)	0.16	0.41
	Right TA	99.51	125.86	−14.85 (−47.38 to 17.69)	0.37	0.62	108.24	115.14	−5.59 (−13.65 to 2.47)	0.17	0.43	101.26	113.12	−10.02 (−21.58 to 1.55)	0.09	0.29
	Left BFs	120.73	115.96	10.40 (−23.35 to 44.16)	0.54	0.81	107.49	117.23	−8.68 (−15.98 to −1.38)	0.02	0.24	102.89	115.35	−10.83 (−20.46 to −1.20)	0.03	0.28
	Right BFs	111.38	124.18	−9.31 (−41.71 to 26.51)	0.57	0.81	109.35	115.52	−5.67 (−13.60 to 2.27)	0.16	0.41	102.78	114.30	−10.80 (−21.82 to 0.23)	0.05	0.29
	Left MG	109.86	114.13	−7.59 (−41.70 to 26.51)	0.66	0.88	114.68	114.64	−1.12 (−9.45 to 7.21)	0.79	0.90	112.39	111.91	−1.35 (−11.57 to 8.88)	0.79	0.90
	Right MG	128.15	124.62	4.73 (−31.53 to 40.98)	0.80	0.90	113.67	121.89	−7.30 (−15.63 to 1.03)	0.09	0.29	112.18	122.72	−9.20 (−20.69 to 2.28)	0.12	0.35
Phase 1	Left RF	113.81	127.67	−18.24 (−47.10 to 10.62)	0.21	0.45	123.76	129.43	−6.33 (−10.56 to −2.11)	<0.001	0.09	124.23	131.88	−8.43 (−14.73 to −2.12)	0.01	0.13
	Right RF	120.63	95.88	19.40 (−10.25 to 49.04)	0.20	0.43	125.46	126.31	−0.82 (−4.79 to 3.14)	0.68	0.89	129.09	127.04	1.95 (−4.79 to 8.70)	0.57	0.81
	Left TA	118.50	135.36	−18.64 (−46.86 to 9.59)	0.19	0.43	123.98	126.54	−2.89 (−6.04 to 0.25)	0.07	0.29	125.10	127.56	−2.50 (−7.47 to 2.47)	0.32	0.57
	Right TA	124.58	117.02	5.38 (−22.75 to 33.50)	0.71	0.89	124.86	126.71	−2.07 (−6.04 to 1.89)	0.30	0.54	125.72	128.10	−2.79 (−8.75 to 3.18)	0.36	0.61
	Left BFs	103.81	106.85	−1.44 (−30.84 to 27.95)	0.92	0.96	123.24	129.54	−6.26 (−12.16 to −0.36)	0.04	0.28	123.36	132.98	−9.96 (−19.03 to −0.89)	0.03	0.28
	Right BFs	110.07	134.04	−27.56 (−58.93 to 3.82)	0.08	0.29	122.68	128.35	−5.60 (−10.99 to −0.22)	0.04	0.28	122.79	131.48	−8.13 (−17.07 to 0.82)	0.07	0.29
	Left MG	132.93	140.07	−4.67 (−41.82 to 32.46)	0.80	0.90	128.63	129.55	−0.24 (−5.60 to 5.12)	0.93	0.96	130.26	130.14	1.38 (−6.52 to 9.28)	0.73	0.89
	Right MG	143.09	141.22	6.00 (−27.40 to 39.39)	0.72	0.89	125.87	127.76	−1.35 (−6.84 to 4.14)	0.63	0.87	126.20	129.39	−1.92 (−10.49 to 6.65)	0.66	0.88
Phase 2	Left RF	79.21	119.51	−44.23 (−70.19 to −18.27)	<0.001	0.07	123.62	125.27	−2.82 (−6.01 to 0.37)	0.08	0.29	119.42	125.04	−7.43 (−12.34 to −2.53)	<0.001	0.09
	Right RF	105.15	125.09	−15.05 (−39.39 to 9.29)	0.22	0.45	129.33	128.07	1.84 (−0.86 to 4.54)	0.18	0.43	128.24	128.46	0.31 (−3.86 to 4.48)	0.88	0.95
	Left TA	123.56	111.81	4.88 (−22.64 to 32.40)	0.73	0.89	135.32	131.29	1.76 (−4.27 to 7.80)	0.56	0.81	139.02	134.23	2.36 (−5.95 to 10.67)	0.58	0.81
	Right TA	114.32	135.38	−23.09 (−45.40 to −0.78)	0.04	0.28	131.31	131.75	−0.05 (−4.66 to 4.56)	0.98	0.98	128.92	133.75	−4.31 (−11.20 to 2.57)	0.22	0.45
	Left BFs	79.76	95.38	−21.15 (−45.59 to 3.30)	0.09	0.29	116.51	122.09	−6.14 (−12.18 to −0.09)	0.05	0.28	108.78	120.36	−12.40 (−20.95 to −3.85)	<0.001	0.09
	Right BFs	128.65	135.98	−3.09 (−29.95 to 23.78)	0.82	0.90	140.02	136.32	4.36 (−1.71 to 10.44)	0.16	0.41	145.27	141.23	4.66 (−3.16 to 12.47)	0.24	0.47
	Left MG	91.14	85.00	1.53 (−29.11 to 32.18)	0.92	0.96	122.41	124.56	−2.31 (−6.47 to 1.84)	0.27	0.52	122.49	124.26	−2.18 (−7.49 to 3.14)	0.42	0.67
	Right MG	115.65	125.11	−14.06 (−46.49 to 18.38)	0.39	0.64	129.84	128.76	0.55 (−4.32 to 5.43)	0.82	0.90	132.52	132.91	−0.90 (−8.79 to 7.00)	0.82	0.90

Values for HCs and PDs are presented as mean ± SD. Adjusted β represents the estimated PDs–HCs difference from multivariable linear regression adjusted for age, sex, and MoCA-K score (HCs = 0, PDs = 1). Negative β values indicate lower temporal indices in PDs. *p*-values correspond to the adjusted group effect, and q-values were obtained using the Benjamini–Hochberg FDR correction across the 72 Grad-CAM comparisons. Significant differences between groups are indicated in *p* < 0.05. No comparison remained statistically significant at q < 0.05. Abbreviations: Grad-CAM, gradient-weighted class activation mapping; PDs, individuals with Parkinson’s disease; HCs, healthy controls; STW, sit-to-walk; sEMG, surface electromyography; RF, rectus femoris; TA, tibialis anterior; BFs, biceps femoris short head; MG, medial gastrocnemius; MaxIdx, maximum attention index; CenterIdx, center of mass index; HalfEnergyIdx, half-energy index.

## Data Availability

The raw data supporting the conclusions of this article will be made available by the authors on request. All data preprocessing, feature extraction, machine learning, deep learning, and explainable artificial intelligence (XAI) analyses were performed in a Conda-managed Python environment. Conventional machine learning analyses were implemented using scikit-learn, imbalanced-learn, and XGBoost, whereas convolutional neural network (CNN) models were developed using TensorFlow (v2.13.0) with the Keras API (v2.13.1). Model interpretability was achieved using SHAP (v0.44.1) for feature importance analysis and Grad-CAM for CNN visualization. Time–frequency representations were generated using PyWavelets (v1.1.1), and data preprocessing and numerical analyses were performed using NumPy (v1.24.4), pandas, and SciPy (v1.10.1). Statistical analyses were supported using statsmodels (v0.14.1). Figures were generated using Matplotlib (v3.7.5) and Seaborn (v0.11.1), and Excel file input/output was managed using Openpyxl (v3.0.7). The analysis code used in this study has been deposited in the Zenodo public repository and is openly available at https://doi.org/10.5281/zenodo.22263874.

## Supplementary Materials

The following supporting information can be downloaded at https://www.mdpi.com/article/10.3390/s26185909/s1, Table S1: The definitions and computational formulas for all EMG-derived variables; Table S2: Univariate between-group comparisons of the sEMG-derived variables; Table S3: Covariate-adjusted between-group comparisons of the sEMG-derived variables; Table S4: Age-matched sensitivity analysis of the leakage-controlled random forest classifier; Table S5: Complete exploratory correlations between sEMG/Grad-CAM-derived measures and clinical scores.

## Abbreviations

The following abbreviations are used in this manuscript:

AP	anteroposterior
APA	anticipatory postural adjustments
AUC	area under the receiver operating characteristic curve
ROC	receiver operating characteristic
BFs	biceps femoris short head
CNN	convolutional neural network
GRAD-CAM	gradient-weighted class activation mapping
HC	healthy control
iEMG	integrated surface electromyography
MaxIdx	maximum attention index
CenterIdx	center of mass index
HalfEnergyIdx	half-energy cumulative index
Ch	channel
MG	medial gastrocnemius
PD	Parkinson’s disease
RF	rectus femoris
SHAP	SHapley Additive exPlanations
SMOTE	synthetic minority oversampling technique
sEMG	surface electromyography
STW	sit-to-walk
TA	tibialis anterior
XAI	explainable artificial intelligence
UPDRS	Unified Parkinson’s Disease Rating Scale
H&Y	Hoehn and Yahr
ML	machine learning
DL	deep learning
RMS	root mean square
SD	standard deviation
LR	logistic regression
SVM	support vector machine with a linear kernel
XGBoost	extreme gradient boosting
CV	cross-validation
