# Supplementary material for: Altered Neuromuscular Time–Frequency Organization During Sit-to-Walk in Parkinson’s Disease: An Explainable Artificial Intelligence Approach Using Surface Electromyography
Source: Sensors (Basel). 2026 Sep 18;26(18):5909. doi: 10.3390/s26185909 (PMC13611564; doi:10.3390/s26185909)
Supplement: Supplementary file 1 [file sensors-26-05909-s001.zip › sensors-4499231-supplementary.pdf]

Table S1. The definitions and computational formulas for all EMG-derived variables

This table organizes the features into several domains. Feature extraction was automated with custom MATLAB scripts.

Features	Definition	Computational formula
Root Mean Square (RMS)	Represents the overall magnitude of the EMG signal, reflecting average muscle activation intensity.	$RMS = \sqrt{\frac{1}{N}\sum_{i=1}^{N}(x_i)^2}$<br>$x_i$: *filtered EMG signal at sample i*<br>N: *number of samples*
Integrated EMG (iEMG)	Cumulative measure of total muscle activity over a period.	$iEMG = \int_0^T \lvert x(t)\rvert dt$<br>$iEMG \approx \sum_{n=1}^{N} \lvert x[n]\rvert \cdot \Delta t$<br>$iEMG \approx \Delta t\left(\frac{\lvert x[1]\rvert}{2} + \sum_{n=2}^{N-1}\lvert x[n]\rvert + \frac{\lvert x[N]\rvert}{2}\right)$<br>$x(t)$: *EMG signal as a function of time*<br>T: *duration of the signal*<br>$x[n]$: *EMG signal at sample n*<br>N: *number of samples*<br>Δt: *sampling interval*
Peak Amplitude	Maximum absolute amplitude of the EMG signal.	$Peak\ Amplitude = \max_{0 \le t \le T} \lvert x(t)\rvert$<br>$x(t)$: *EMG signal as a function of time*<br>T: *duration of the signal*
Co-contraction Index (CCI)	Degree of simultaneous activation between agonist–antagonist muscle pairs (e.g., RF–BFs, TA–MG).	$x_{norm}(t) = \frac{x(t)}{\max(\lvert x(t)\rvert)}$<br>$CCI = \frac{\sum_t(\lvert EMGa_{norm}(t)\rvert \cdot \lvert EMGan_{norm}(t)\rvert)}{\sum_t(\lvert EMGa_{norm}(t)\rvert + \lvert EMGan_{norm}(t)\rvert)}$<br>$x(t)$: *EMG signal at time t*<br>$x_{norm}(t)$: *normalized EMG signal*<br>$\max(\lvert x(t)\rvert)$: *maximum absolute amplitude of the EMG signal over the entire duration*<br>$EMGa_{norm}(t)$: *normalized agonist EMG signal*<br>$EMGan_{norm}(t)$: *normalized antagonist EMG signal*
Median Frequency	Frequency below which 50% of the total EMG power spectrum is contained.	$x(f) = FFT\{x(t)\}$<br>$P(f) = \left\lvert\frac{X(f)}{N}\right\rvert$<br>$C(f) = \sum_{i=0}^{k} P(f_i)$<br>$P_{total} = C(f_{max})$<br>$f_{med}\ such\ that\ C(f_{med}) \ge \frac{P_{total}}{2}$

		$x(t)$: EMG signal in the time domain<br>$X(f)$: Fourier transform of $x(t)$<br>$x(t)$: EMG signal in the time domain<br>N: number of samples<br>$P(f)$: amplitude spectrum (magnitude normalized by N)<br>$C(f_k)$: cumulative sum of spectral power up to frequency bin f_k<br>P_{total}: cumulative spectral power across all frequency bins
Mean Frequency	Power-weighted average frequency of the EMG spectrum.	$f_{mean} = \frac{\sum_{i=1}^{N} f_i \cdot P(f_i)}{\sum_{i=1}^{N} P(f_i)}$<br>$f_i$: frequency at the ith bin<br>$P(f_i)$: power (or amplitude spectrum)at frequency f_i<br>N: total number of frequency bins
Frequency Band Power (20–150 Hz)	Power of EMG within a specific frequency range, reflecting muscle recruitment dynamics.	$P_{band} = \sum_{f=f_{20}}^{f_{150}} P(f)$<br>$P(f)$: power spectrum at frequency f<br>f_{20}: lower cutoff frequency (20 Hz)<br>f_{150}: upper cutoff frequency (150 Hz)
Entropy	Complexity and irregularity of the EMG frequency spectrum.	$H = -\sum_{i=1}^{N} P(f_i) \log(P(f_i) + \epsilon)$<br>$P(f_i)$: power spectrum at frequency bin f_i<br>N: total number of frequency bins<br>ϵ: small constant to avoid taking the logarithm of zero
AP SD	The standard deviation (SD) of the EMG signal in the anteroposterior directions, quantifying variability in muscle activation, reflecting postural stability.	$AP\_SD = \sqrt{\frac{1}{N-1}\sum_{i=1}^{N}(x_i - \bar{x})^2}$<br>$x_i$: anteroposterior displacement at sample i<br>$\bar{x}$: mean values of AP displacement<br>N: number of samples
ML SD	The SD of the EMG signal in the mediolateral directions, quantifying variability in muscle activation, reflecting lateral stability.	$ML\_SD = \sqrt{\frac{1}{N-1}\sum_{i=1}^{N}(y_i - \bar{y})^2}$<br>$y_i$: midiolateral displacement at sample i<br>$\bar{y}$: mean values of ML displacement<br>N: number of samples

Table S2. Univariate between-group comparisons of the sEMG-derived variables

STW phase	sEMG variable	HC (n=50)	PD (n=101)	Statistical test	Raw p-value	FDR q-value
Phase 1	EMG1 RMS	1.118e-05 ± 6.699e-06	1.153e-05 ± 7.023e-06	Mann–Whitney U	0.913	0.942
Phase 1	EMG2 RMS	1.199e-05 ± 9.420e-06	1.185e-05 ± 6.829e-06	Mann–Whitney U	0.489	0.768
Phase 1	EMG3 RMS	5.267e-05 ± 2.116e-04	2.177e-05 ± 3.487e-05	Mann–Whitney U	0.898	0.942
Phase 1	EMG4 RMS	6.681e-05 ± 2.845e-04	1.878e-05 ± 2.469e-05	Mann–Whitney U	0.950	0.964
Phase 1	EMG5 RMS	8.779e-06 ± 5.366e-06	1.049e-05 ± 1.227e-05	Mann–Whitney U	0.185	0.406
Phase 1	EMG6 RMS	9.676e-06 ± 3.279e-06	1.181e-05 ± 1.723e-05	Mann–Whitney U	0.981	0.981
Phase 1	EMG7 RMS	7.975e-06 ± 9.839e-06	1.381e-05 ± 6.871e-05	Mann–Whitney U	0.737	0.908
Phase 1	EMG8 RMS	7.350e-06 ± 7.225e-06	3.078e-05 ± 1.168e-04	Mann–Whitney U	0.723	0.908
Phase 1	Integrated EMG1	9.365e-04 ± 6.914e-04	0.00121 ± 0.00055	Mann–Whitney U	0.001	0.009
Phase 1	Integrated EMG2	9.766e-04 ± 7.507e-04	0.00124 ± 0.00053	Mann–Whitney U	0.000	0.002
Phase 1	Integrated EMG3	0.00413 ± 0.01636	0.00229 ± 0.00334	Mann–Whitney U	0.035	0.127
Phase 1	Integrated EMG4	0.00554 ± 0.02553	0.00190 ± 0.00175	Mann–Whitney U	0.048	0.150
Phase 1	Integrated EMG5	7.585e-04 ± 4.260e-04	0.00106 ± 0.00057	Mann–Whitney U	0.000	0.000
Phase 1	Integrated EMG6	8.746e-04 ± 3.304e-04	0.00118 ± 0.00068	Mann–Whitney U	0.000	0.002
Phase 1	Integrated EMG7	0.00077 ± 0.00110	0.00173 ± 0.00818	Mann–Whitney U	0.027	0.105
Phase 1	Integrated EMG8	6.796e-04 ± 7.626e-04	0.00344 ± 0.01315	Mann–Whitney U	0.022	0.091
Phase 1	Peak Amplitude EMG1	4.690e-05 ± 3.309e-05	5.085e-05 ± 3.587e-05	Mann–Whitney U	0.568	0.824
Phase 1	Peak Amplitude EMG2	5.180e-05 ± 5.149e-05	5.099e-05 ± 3.252e-05	Mann–Whitney U	0.256	0.490
Phase 1	Peak Amplitude EMG3	0.00025 ± 0.00108	9.298e-05 ± 1.751e-04	Mann–Whitney U	0.719	0.908
Phase 1	Peak Amplitude EMG4	0.00030 ± 0.00126	7.873e-05 ± 1.186e-04	Mann–Whitney U	0.863	0.942
Phase 1	Peak Amplitude EMG5	3.585e-05 ± 2.560e-05	4.566e-05 ± 5.481e-05	Mann–Whitney U	0.048	0.150
Phase 1	Peak Amplitude EMG6	3.786e-05 ± 1.316e-05	5.188e-05 ± 7.632e-05	Mann–Whitney U	0.267	0.490
Phase 1	Peak Amplitude EMG7	3.610e-05 ± 6.351e-05	6.422e-05 ± 3.395e-04	Mann–Whitney U	0.391	0.679
Phase 1	Peak Amplitude EMG8	3.143e-05 ± 3.949e-05	1.713e-04 ± 6.653e-04	Mann–Whitney U	0.106	0.279
Phase 1	Co-contraction IndexL	0.115 ± 0.024	0.09738 ± 0.02892	Mann–Whitney U	0.000	0.000
Phase 1	Co-contraction IndexR	0.108 ± 0.027	0.09535 ± 0.03071	Mann–Whitney U	0.006	0.036
Phase 1	Median Frequency	306.57 ± 33.64	325.37 ± 26.90	Mann–Whitney U	0.000	0.003
Phase 1	Mean Frequency	311.96 ± 29.06	327.72 ± 22.74	Mann–Whitney U	0.002	0.011
Phase 1	Frequency Band Power	5.105e-06 ± 2.930e-06	5.424e-06 ± 5.892e-06	Mann–Whitney U	0.549	0.824
Phase 1	Entropy	5.291e-04 ± 3.035e-04	6.119e-04 ± 2.858e-04	Mann–Whitney U	0.152	0.345
Phase 1	AP SD	1.109e-05 ± 6.544e-06	1.163e-05 ± 7.023e-06	Mann–Whitney U	0.840	0.942
Phase 1	ML SD	1.218e-05 ± 9.573e-06	1.175e-05 ± 6.999e-06	Mann–Whitney U	0.599	0.824
Phase 2	EMG1 RMS	1.481e-05 ± 1.036e-05	1.235e-05 ± 6.681e-06	Mann–Whitney U	0.596	0.824
Phase 2	EMG2 RMS	1.708e-05 ± 1.106e-05	2.060e-05 ± 1.377e-05	Mann–Whitney U	0.098	0.268
Phase 2	EMG3 RMS	1.546e-04 ± 6.652e-04	6.707e-05 ± 1.909e-04	Mann–Whitney U	0.552	0.824
Phase 2	EMG4 RMS	1.575e-04 ± 6.922e-04	8.547e-05 ± 3.178e-04	Mann–Whitney U	0.222	0.457
Phase 2	EMG5 RMS	1.201e-05 ± 7.099e-06	1.116e-05 ± 4.625e-06	Mann–Whitney U	0.752	0.908
Phase 2	EMG6 RMS	1.288e-05 ± 4.960e-06	1.485e-05 ± 6.412e-06	Mann–Whitney U	0.020	0.086
Phase 2	EMG7 RMS	3.489e-05 ± 4.652e-05	6.007e-05 ± 1.828e-04	Mann–Whitney U	0.142	0.341
Phase 2	EMG8 RMS	2.661e-05 ± 3.580e-05	1.492e-04 ± 3.948e-04	Mann–Whitney U	0.000	0.002
Phase 2	Integrated EMG1	0.00110 ± 0.00078	9.617e-04 ± 4.766e-04	Mann–Whitney U	0.913	0.942
Phase 2	Integrated EMG2	0.00122 ± 0.00079	0.00137 ± 0.00077	Mann–Whitney U	0.124	0.314
Phase 2	Integrated EMG3	0.01335 ± 0.06373	0.00434 ± 0.01069	Mann–Whitney U	0.352	0.628
Phase 2	Integrated EMG4	0.01388 ± 0.07048	0.00553 ± 0.02039	Mann–Whitney U	0.264	0.490
Phase 2	Integrated EMG5	9.226e-04 ± 5.153e-04	9.303e-04 ± 4.294e-04	Mann–Whitney U	0.257	0.490
Phase 2	Integrated EMG6	0.00100 ± 0.00039	0.00117 ± 0.00045	Mann–Whitney U	0.003	0.019
Phase 2	Integrated EMG7	0.00235 ± 0.00299	0.00436 ± 0.01410	Mann–Whitney U	0.054	0.163
Phase 2	Integrated EMG8	0.00194 ± 0.00265	0.00994 ± 0.02678	Mann–Whitney U	0.000	0.002
Phase 2	Peak Amplitude EMG1	5.754e-05 ± 4.540e-05	4.969e-05 ± 3.449e-05	Mann–Whitney U	0.899	0.942
Phase 2	Peak Amplitude EMG2	6.779e-05 ± 4.434e-05	9.098e-05 ± 6.626e-05	Mann–Whitney U	0.037	0.128
Phase 2	Peak Amplitude EMG3	0.00049 ± 0.00187	3.051e-04 ± 8.814e-04	Mann–Whitney U	0.451	0.744
Phase 2	Peak Amplitude EMG4	0.00052 ± 0.00184	0.00037 ± 0.00102	Mann–Whitney U	0.081	0.233
Phase 2	Peak Amplitude EMG5	4.463e-05 ± 3.142e-05	3.961e-05 ± 1.931e-05	Mann–Whitney U	0.780	0.920
Phase 2	Peak Amplitude EMG6	4.806e-05 ± 2.313e-05	5.754e-05 ± 2.968e-05	Mann–Whitney U	0.007	0.038
Phase 2	Peak Amplitude EMG7	1.434e-04 ± 1.976e-04	2.334e-04 ± 6.745e-04	Mann–Whitney U	0.220	0.457
Phase 2	Peak Amplitude EMG8	1.095e-04 ± 1.688e-04	0.00061 ± 0.00159	Mann–Whitney U	0.000	0.002
Phase 2	Co-contraction IndexL_1	0.118 ± 0.017	0.110 ± 0.022	Mann–Whitney U	0.020	0.086
Phase 2	Co-contraction IndexR_1	0.113 ± 0.018	0.112 ± 0.020	Mann–Whitney U	0.873	0.942
Phase 2	Co-contraction IndexL_2	0.104 ± 0.023	0.105 ± 0.023	Mann–Whitney U	0.656	0.884
Phase 2	Co-contraction IndexR_2	0.108 ± 0.021	0.105 ± 0.021	Independent-samples t-test	0.462	0.744
Phase 2	Median Frequency	297.13 ± 27.02	295.49 ± 26.94	Mann–Whitney U	0.674	0.889
Phase 2	Mean Frequency	303.34 ± 21.12	299.84 ± 22.69	Mann–Whitney U	0.416	0.705
Phase 2	Frequency Band Power	6.715e-06 ± 5.105e-06	5.907e-06 ± 4.574e-06	Mann–Whitney U	0.825	0.942
Phase 2	Entropy	6.098e-04 ± 3.928e-04	5.378e-04 ± 2.671e-04	Mann–Whitney U	0.756	0.908
Phase 2	AP SD	1.425e-05 ± 9.979e-06	1.204e-05 ± 6.190e-06	Mann–Whitney U	0.588	0.824
Phase 2	ML SD	1.778e-05 ± 1.135e-05	2.105e-05 ± 1.384e-05	Mann–Whitney U	0.145	0.341

Note. Data are presented descriptively as mean ± SD. Normality was assessed using the Shapiro–Wilk test. An independent-samples t-test was used when both groups met the normality criterion; otherwise, the Mann–Whitney U test was used. Benjamini–Hochberg false discovery rate (FDR) correction was applied across the 66 sEMG comparisons. HC, healthy control; PD, Parkinson's disease; sEMG, surface electromyography.

Table S3. Covariate-adjusted between-group comparisons of the sEMG-derived variables

STW phase	sEMG variable	HC (n=50)	PD (n=101)	Adjusted β (PD−HCs)	95% CI lower	95% CI upper	Raw p-value	FDR q-value
Phase 1	EMG1 RMS	1.118e-05 ± 6.699e-06	1.153e-05 ± 7.023e-06	4.912E-07	-2.005E-06	2.988E-06	0.698	0.793
Phase 1	EMG2 RMS	1.199e-05 ± 9.420e-06	1.185e-05 ± 6.829e-06	-1.766E-07	-2.983E-06	2.630E-06	0.901	0.929
Phase 1	EMG3 RMS	5.267e-05 ± 2.116e-04	2.177e-05 ± 3.487e-05	-2.439E-05	-6.919E-05	2.041E-05	0.284	0.616
Phase 1	EMG4 RMS	6.681e-05 ± 2.845e-04	1.878e-05 ± 2.469e-05	-4.119E-05	-1.004E-04	1.801E-05	0.171	0.538
Phase 1	EMG5 RMS	8.779e-06 ± 5.366e-06	1.049e-05 ± 1.227e-05	1.386E-06	-2.394E-06	5.166E-06	0.470	0.669
Phase 1	EMG6 RMS	9.676e-06 ± 3.279e-06	1.181e-05 ± 1.723e-05	1.595E-06	-3.520E-06	6.710E-06	0.539	0.711
Phase 1	EMG7 RMS	7.975e-06 ± 9.839e-06	1.381e-05 ± 6.871e-05	2.136E-06	-1.815E-05	2.242E-05	0.835	0.889
Phase 1	EMG8 RMS	7.350e-06 ± 7.225e-06	3.078e-05 ± 1.168e-04	1.569E-05	-1.834E-05	4.973E-05	0.364	0.616
Phase 1	Integrated EMG1	9.365e-04 ± 6.914e-04	0.00121 ± 0.00055	2.629E-04	4.651E-05	4.794E-04	0.018	0.145
Phase 1	Integrated EMG2	9.766e-04 ± 7.507e-04	0.00124 ± 0.00053	2.583E-04	3.861E-05	4.779E-04	0.022	0.158
Phase 1	Integrated EMG3	0.00413 ± 0.01636	0.00229 ± 0.00334	-1.416E-03	-4.934E-03	2.102E-03	0.428	0.639
Phase 1	Integrated EMG4	0.00554 ± 0.02553	0.00190 ± 0.00175	-3.130E-03	-8.429E-03	2.170E-03	0.245	0.616
Phase 1	Integrated EMG5	7.585e-04 ± 4.260e-04	0.00106 ± 0.00057	2.730E-04	8.423E-05	4.617E-04	0.005	0.081
Phase 1	Integrated EMG6	8.746e-04 ± 3.304e-04	0.00118 ± 0.00068	2.672E-04	5.720E-05	4.772E-04	0.013	0.145
Phase 1	Integrated EMG7	0.00077 ± 0.00110	0.00173 ± 0.00818	4.769E-04	-1.933E-03	2.887E-03	0.696	0.793
Phase 1	Integrated EMG8	6.796e-04 ± 7.626e-04	0.00344 ± 0.01315	1.653E-03	-2.151E-03	5.456E-03	0.392	0.616
Phase 1	Peak Amplitude EMG1	4.690e-05 ± 3.309e-05	5.085e-05 ± 3.587e-05	5.566E-06	-7.025E-06	1.816E-05	0.384	0.616
Phase 1	Peak Amplitude EMG2	5.180e-05 ± 5.149e-05	5.099e-05 ± 3.252e-05	-1.854E-08	-1.437E-05	1.434E-05	0.998	0.998
Phase 1	Peak Amplitude EMG3	0.00025 ± 0.00108	9.298e-05 ± 1.751e-04	-1.207E-04	-3.482E-04	1.067E-04	0.296	0.616
Phase 1	Peak Amplitude EMG4	0.00030 ± 0.00126	7.873e-05 ± 1.186e-04	-1.864E-04	-4.492E-04	7.639E-05	0.163	0.538
Phase 1	Peak Amplitude EMG5	3.585e-05 ± 2.560e-05	4.566e-05 ± 5.481e-05	8.395E-06	-8.623E-06	2.541E-05	0.331	0.616
Phase 1	Peak Amplitude EMG6	3.786e-05 ± 1.316e-05	5.188e-05 ± 7.632e-05	1.135E-05	-1.129E-05	3.399E-05	0.323	0.616
Phase 1	Peak Amplitude EMG7	3.610e-05 ± 6.351e-05	6.422e-05 ± 3.395e-04	9.171E-06	-9.138E-05	1.097E-04	0.857	0.898
Phase 1	Peak Amplitude EMG8	3.143e-05 ± 3.949e-05	1.713e-04 ± 6.653e-04	9.081E-05	-1.025E-04	2.841E-04	0.355	0.616
Phase 1	Co-contraction IndexL	0.115 ± 0.024	0.09738 ± 0.02892	-0.019	-0.029	-0.009	0.000	0.010
Phase 1	Co-contraction IndexR	0.108 ± 0.027	0.09535 ± 0.03071	-0.013	-0.024	-0.002	0.017	0.145
Phase 1	Median Frequency	306.57 ± 33.64	325.37 ± 26.90	19.259	8.799	29.718	0.000	0.013
Phase 1	Mean Frequency	311.96 ± 29.06	327.72 ± 22.74	15.685	6.766	24.604	0.001	0.015
Phase 1	Frequency Band Power	5.105e-06 ± 2.930e-06	5.424e-06 ± 5.892e-06	5.236E-07	-1.313E-06	2.360E-06	0.574	0.720
Phase 1	Entropy	5.291e-04 ± 3.035e-04	6.119e-04 ± 2.858e-04	8.536E-05	-1.975E-05	1.905E-04	0.111	0.425
Phase 1	AP SD	1.109e-05 ± 6.544e-06	1.163e-05 ± 7.023e-06	6.405E-07	-1.839E-06	3.120E-06	0.610	0.733
Phase 1	ML SD	1.218e-05 ± 9.573e-06	1.175e-05 ± 6.999e-06	-4.438E-07	-3.305E-06	2.417E-06	0.760	0.836
Phase 2	EMG1 RMS	1.481e-05 ± 1.036e-05	1.235e-05 ± 6.681e-06	-2.333E-06	-5.249E-06	5.828E-07	0.116	0.425
Phase 2	EMG2 RMS	1.708e-05 ± 1.106e-05	2.060e-05 ± 1.377e-05	2.802E-06	-1.737E-06	7.340E-06	0.224	0.616
Phase 2	EMG3 RMS	1.546e-04 ± 6.652e-04	6.707e-05 ± 1.909e-04	-6.611E-05	-2.144E-04	8.218E-05	0.380	0.616
Phase 2	EMG4 RMS	1.575e-04 ± 6.922e-04	8.547e-05 ± 3.178e-04	-7.608E-05	-2.472E-04	9.499E-05	0.381	0.616
Phase 2	EMG5 RMS	1.201e-05 ± 7.099e-06	1.116e-05 ± 4.625e-06	-8.102E-07	-2.794E-06	1.174E-06	0.421	0.639
Phase 2	EMG6 RMS	1.288e-05 ± 4.960e-06	1.485e-05 ± 6.412e-06	1.835E-06	-2.533E-07	3.924E-06	0.085	0.349
Phase 2	EMG7 RMS	3.489e-05 ± 4.652e-05	6.007e-05 ± 1.828e-04	1.626E-05	-3.840E-05	7.092E-05	0.557	0.720
Phase 2	EMG8 RMS	2.661e-05 ± 3.580e-05	1.492e-04 ± 3.948e-04	1.198E-04	3.676E-06	2.360E-04	0.043	0.215
Phase 2	Integrated EMG1	0.00110 ± 0.00078	9.617e-04 ± 4.766e-04	-1.197E-04	-3.331E-04	9.362E-05	0.269	0.616
Phase 2	Integrated EMG2	0.00122 ± 0.00079	0.00137 ± 0.00077	1.522E-04	-1.247E-04	4.292E-04	0.279	0.616
Phase 2	Integrated EMG3	0.01335 ± 0.06373	0.00434 ± 0.01069	-0.007	-0.020	0.007	0.311	0.616
Phase 2	Integrated EMG4	0.01388 ± 0.07048	0.00553 ± 0.02039	-0.008	-0.024	0.008	0.330	0.616
Phase 2	Integrated EMG5	9.226e-04 ± 5.153e-04	9.303e-04 ± 4.294e-04	3.119E-05	-1.336E-04	1.960E-04	0.709	0.793
Phase 2	Integrated EMG6	0.00100 ± 0.00039	0.00117 ± 0.00045	1.918E-04	3.893E-05	3.446E-04	0.014	0.145
Phase 2	Integrated EMG7	0.00235 ± 0.00299	0.00436 ± 0.01410	1.404E-03	-2.795E-03	5.604E-03	0.510	0.687
Phase 2	Integrated EMG8	0.00194 ± 0.00265	0.00994 ± 0.02678	0.008	0.000	0.016	0.043	0.215
Phase 2	Peak Amplitude EMG1	5.754e-05 ± 4.540e-05	4.969e-05 ± 3.449e-05	-5.465E-06	-1.927E-05	8.344E-06	0.435	0.639
Phase 2	Peak Amplitude EMG2	6.779e-05 ± 4.434e-05	9.098e-05 ± 6.626e-05	2.059E-05	-4.920E-07	4.167E-05	0.056	0.244
Phase 2	Peak Amplitude EMG3	0.00049 ± 0.00187	3.051e-04 ± 8.814e-04	-1.050E-04	-5.694E-04	3.593E-04	0.656	0.773
Phase 2	Peak Amplitude EMG4	0.00052 ± 0.00184	0.00037 ± 0.00102	-1.647E-04	-6.507E-04	3.212E-04	0.504	0.687
Phase 2	Peak Amplitude EMG5	4.463e-05 ± 3.142e-05	3.961e-05 ± 1.931e-05	-4.187E-06	-1.273E-05	4.357E-06	0.334	0.616
Phase 2	Peak Amplitude EMG6	4.806e-05 ± 2.313e-05	5.754e-05 ± 2.968e-05	9.825E-06	1.940E-07	1.946E-05	0.046	0.215
Phase 2	Peak Amplitude EMG7	1.434e-04 ± 1.976e-04	2.334e-04 ± 6.745e-04	5.666E-05	-1.461E-04	2.594E-04	0.582	0.720
Phase 2	Peak Amplitude EMG8	1.095e-04 ± 1.688e-04	0.00061 ± 0.00159	5.087E-04	3.984E-05	9.776E-04	0.034	0.212
Phase 2	Co-contraction IndexL_1	0.118 ± 0.017	0.110 ± 0.022	-0.008	-0.016	-0.001	0.035	0.212
Phase 2	Co-contraction IndexR_1	0.113 ± 0.018	0.112 ± 0.020	-2.476E-03	-9.328E-03	4.376E-03	0.476	0.669
Phase 2	Co-contraction IndexL_2	0.104 ± 0.023	0.105 ± 0.023	5.142E-05	-8.153E-03	8.256E-03	0.990	0.998
Phase 2	Co-contraction IndexR_2	0.108 ± 0.021	0.105 ± 0.021	-0.004	-0.012	0.004	0.306	0.616
Phase 2	Median Frequency	297.13 ± 27.02	295.49 ± 26.94	-1.241	-10.703	8.222	0.796	0.861
Phase 2	Mean Frequency	303.34 ± 21.12	299.84 ± 22.69	-3.565	-11.480	4.349	0.375	0.616
Phase 2	Frequency Band Power	6.715e-06 ± 5.105e-06	5.907e-06 ± 4.574e-06	-4.641E-07	-2.158E-06	1.230E-06	0.589	0.720
Phase 2	Entropy	6.098e-04 ± 3.928e-04	5.378e-04 ± 2.671e-04	-5.607E-05	-1.691E-04	5.697E-05	0.329	0.616
Phase 2	AP SD	1.425e-05 ± 9.979e-06	1.204e-05 ± 6.190e-06	-2.112E-06	-4.869E-06	6.448E-07	0.132	0.459
Phase 2	ML SD	1.778e-05 ± 1.135e-05	2.105e-05 ± 1.384e-05	2.576E-06	-2.015E-06	7.166E-06	0.269	0.616

Note. HC and PD values are presented descriptively as mean ± SD. Adjusted between-group effects were estimated using multivariable linear regression with group (HCs=0, PD=1) as the primary independent variable and age, sex, and MoCA-K score as covariates. Adjusted β represents the estimated PD − HCs difference after covariate adjustment. Benjamini–Hochberg FDR correction was applied across the 66 sEMG outcomes; q<0.05 was considered statistically significant.

Table S4. Age-matched sensitivity analysis of the leakage-controlled random forest classifier

A. Covariate balance in the age-matched cohort (49 PD–HCs pairs; N=98)

Variable	HC, mean ± SD	PD, mean ± SD	Welch p-value	SMD
Age	65.55 ± 5.47	65.78 ± 5.34	0.838	0.042
MoCA	24.88 ± 3.02	26.27 ± 2.95	0.024	0.465

B. Five-fold random forest performance in the age-matched cohort

Fold	Accuracy (%)	Precision (%)	Sensitivity (%)	Specificity (%)	F1-score (%)	AUC
1	65.00	61.54	80.00	50.00	69.57	0.840
2	75.00	69.23	90.00	60.00	78.26	0.830
3	70.00	70.00	70.00	70.00	70.00	0.790
4	73.68	70.00	77.78	70.00	73.68	0.767
5	84.21	100.00	70.00	100.00	82.35	0.861
Mean ± SD	73.58 ± 7.10	74.15 ± 14.88	77.56 ± 8.29	70.00 ± 18.71	74.77 ± 5.49	0.818 ± 0.038

C. Overall age-matched sensitivity-analysis summary

**Matched sample size (N)**	98.00
**HC / PD, n**	49 / 49
**Age-matching caliper (years)**	1.34
**Mean absolute pair age difference (years)**	0.31
**Maximum pair age difference (years)**	1.00
**RF accuracy, mean ± SD (%)**	73.58 ± 7.10
**RF precision, mean (%)**	74.15
**RF sensitivity, mean (%)**	77.56
**RF specificity, mean (%)**	70.00
**RF F1-score, mean (%)**	74.77
**RF AUC, mean ± SD**	0.818 ± 0.038
**Pooled OOF AUC**	0.81
**OOF confusion matrix (TN, FP, FN, TP)**	34, 15, 11, 38

Note. Age matching used 1:1 matching without replacement with an age caliper of 0.2 pooled SD. The resulting cohort included 49 HC–PDs pairs. SMD, standardized mean difference; RF, random forest; OOF, out-of-fold; AUC, area under the receiver operating characteristic curve; TN, true negative; FP, false positive; FN, false negative; TP, true positive. Matching targeted age only; the MoCA-K imbalance therefore remained and should not be interpreted as balanced by the matching procedure.

Table S5. Complete exploratory correlations between sEMG/Grad-CAM-derived measures and clinical scores

Analysis type	STW phase	Channel / muscle	sEMG or Grad-CAM measure	Clinical score	n	Spearman ρ	Unadjusted p-value
sEMG	Phase 1	Ch1 (Left RF)	EMG1 RMS	H&Y stage	101	0.017	0.865
sEMG	Phase 1	Ch1 (Left RF)	EMG1 RMS	UPDRS Part III	101	-0.029	0.773
sEMG	Phase 1	Ch1 (Left RF)	EMG1 RMS	UPDRS total	101	-0.141	0.158
sEMG	Phase 1	Ch2 (Right RF)	EMG2 RMS	H&Y stage	101	-0.043	0.673
sEMG	Phase 1	Ch2 (Right RF)	EMG2 RMS	UPDRS Part III	101	-0.074	0.461
sEMG	Phase 1	Ch2 (Right RF)	EMG2 RMS	UPDRS total	101	-0.184	0.065
sEMG	Phase 1	Ch3 (Left TA)	EMG3 RMS	H&Y stage	101	-0.041	0.686
sEMG	Phase 1	Ch3 (Left TA)	EMG3 RMS	UPDRS Part III	101	0.011	0.912
sEMG	Phase 1	Ch3 (Left TA)	EMG3 RMS	UPDRS total	101	-0.060	0.548
sEMG	Phase 1	Ch4 (Right TA)	EMG4 RMS	H&Y stage	101	0.162	0.106
sEMG	Phase 1	Ch4 (Right TA)	EMG4 RMS	UPDRS Part III	101	0.174	0.082
sEMG	Phase 1	Ch4 (Right TA)	EMG4 RMS	UPDRS total	101	0.099	0.323
sEMG	Phase 1	Ch5 (Left BF)	EMG5 RMS	H&Y stage	101	0.228	0.022
sEMG	Phase 1	Ch5 (Left BF)	EMG5 RMS	UPDRS Part III	101	0.123	0.220
sEMG	Phase 1	Ch5 (Left BF)	EMG5 RMS	UPDRS total	101	0.120	0.233
sEMG	Phase 1	Ch6 (Right BF)	EMG6 RMS	H&Y stage	101	0.110	0.273
sEMG	Phase 1	Ch6 (Right BF)	EMG6 RMS	UPDRS Part III	101	0.155	0.121
sEMG	Phase 1	Ch6 (Right BF)	EMG6 RMS	UPDRS total	101	0.087	0.384
sEMG	Phase 1	Ch7 (Left MG)	EMG7 RMS	H&Y stage	101	0.012	0.907
sEMG	Phase 1	Ch7 (Left MG)	EMG7 RMS	UPDRS Part III	101	-0.007	0.944
sEMG	Phase 1	Ch7 (Left MG)	EMG7 RMS	UPDRS total	101	0.061	0.547
sEMG	Phase 1	Ch8 (Right MG)	EMG8 RMS	H&Y stage	101	0.059	0.555
sEMG	Phase 1	Ch8 (Right MG)	EMG8 RMS	UPDRS Part III	101	-0.137	0.171
sEMG	Phase 1	Ch8 (Right MG)	EMG8 RMS	UPDRS total	101	-0.045	0.652
sEMG	Phase 1	Ch1 (Left RF)	Integrated EMG1	H&Y stage	101	0.102	0.308
sEMG	Phase 1	Ch1 (Left RF)	Integrated EMG1	UPDRS Part III	101	-0.069	0.494
sEMG	Phase 1	Ch1 (Left RF)	Integrated EMG1	UPDRS total	101	-0.055	0.588
sEMG	Phase 1	Ch2 (Right RF)	Integrated EMG2	H&Y stage	101	0.125	0.213
sEMG	Phase 1	Ch2 (Right RF)	Integrated EMG2	UPDRS Part III	101	-0.055	0.585
sEMG	Phase 1	Ch2 (Right RF)	Integrated EMG2	UPDRS total	101	-0.039	0.702
sEMG	Phase 1	Ch3 (Left TA)	Integrated EMG3	H&Y stage	101	0.018	0.861
sEMG	Phase 1	Ch3 (Left TA)	Integrated EMG3	UPDRS Part III	101	0.030	0.766
sEMG	Phase 1	Ch3 (Left TA)	Integrated EMG3	UPDRS total	101	0.054	0.594
sEMG	Phase 1	Ch4 (Right TA)	Integrated EMG4	H&Y stage	101	0.230	0.021
sEMG	Phase 1	Ch4 (Right TA)	Integrated EMG4	UPDRS Part III	101	0.162	0.105
sEMG	Phase 1	Ch4 (Right TA)	Integrated EMG4	UPDRS total	101	0.182	0.068
sEMG	Phase 1	Ch5 (Left BF)	Integrated EMG5	H&Y stage	101	0.231	0.020
sEMG	Phase 1	Ch5 (Left BF)	Integrated EMG5	UPDRS Part III	101	0.091	0.367
sEMG	Phase 1	Ch5 (Left BF)	Integrated EMG5	UPDRS total	101	0.171	0.088
sEMG	Phase 1	Ch6 (Right BF)	Integrated EMG6	H&Y stage	101	0.075	0.459
sEMG	Phase 1	Ch6 (Right BF)	Integrated EMG6	UPDRS Part III	101	0.071	0.481
sEMG	Phase 1	Ch6 (Right BF)	Integrated EMG6	UPDRS total	101	0.107	0.286
sEMG	Phase 1	Ch7 (Left MG)	Integrated EMG7	H&Y stage	101	0.022	0.824
sEMG	Phase 1	Ch7 (Left MG)	Integrated EMG7	UPDRS Part III	101	-0.021	0.838
sEMG	Phase 1	Ch7 (Left MG)	Integrated EMG7	UPDRS total	101	0.108	0.281
sEMG	Phase 1	Ch8 (Right MG)	Integrated EMG8	H&Y stage	101	0.070	0.486
sEMG	Phase 1	Ch8 (Right MG)	Integrated EMG8	UPDRS Part III	101	-0.138	0.170
sEMG	Phase 1	Ch8 (Right MG)	Integrated EMG8	UPDRS total	101	-0.006	0.956
sEMG	Phase 1	Ch1 (Left RF)	Peak Amplitude EMG1	H&Y stage	101	0.031	0.757
sEMG	Phase 1	Ch1 (Left RF)	Peak Amplitude EMG1	UPDRS Part III	101	-0.007	0.945
sEMG	Phase 1	Ch1 (Left RF)	Peak Amplitude EMG1	UPDRS total	101	-0.121	0.227
sEMG	Phase 1	Ch2 (Right RF)	Peak Amplitude EMG2	H&Y stage	101	0.000	0.999
sEMG	Phase 1	Ch2 (Right RF)	Peak Amplitude EMG2	UPDRS Part III	101	-0.029	0.773
sEMG	Phase 1	Ch2 (Right RF)	Peak Amplitude EMG2	UPDRS total	101	-0.129	0.198
sEMG	Phase 1	Ch3 (Left TA)	Peak Amplitude EMG3	H&Y stage	101	-0.009	0.929
sEMG	Phase 1	Ch3 (Left TA)	Peak Amplitude EMG3	UPDRS Part III	101	0.020	0.844
sEMG	Phase 1	Ch3 (Left TA)	Peak Amplitude EMG3	UPDRS total	101	-0.015	0.880
sEMG	Phase 1	Ch4 (Right TA)	Peak Amplitude EMG4	H&Y stage	101	0.140	0.164
sEMG	Phase 1	Ch4 (Right TA)	Peak Amplitude EMG4	UPDRS Part III	101	0.113	0.262
sEMG	Phase 1	Ch4 (Right TA)	Peak Amplitude EMG4	UPDRS total	101	0.074	0.460
sEMG	Phase 1	Ch5 (Left BF)	Peak Amplitude EMG5	H&Y stage	101	0.254	0.010
sEMG	Phase 1	Ch5 (Left BF)	Peak Amplitude EMG5	UPDRS Part III	101	0.106	0.290
sEMG	Phase 1	Ch5 (Left BF)	Peak Amplitude EMG5	UPDRS total	101	0.136	0.175
sEMG	Phase 1	Ch6 (Right BF)	Peak Amplitude EMG6	H&Y stage	101	0.063	0.529
sEMG	Phase 1	Ch6 (Right BF)	Peak Amplitude EMG6	UPDRS Part III	101	0.176	0.079
sEMG	Phase 1	Ch6 (Right BF)	Peak Amplitude EMG6	UPDRS total	101	0.104	0.300
sEMG	Phase 1	Ch7 (Left MG)	Peak Amplitude EMG7	H&Y stage	101	-0.015	0.884
sEMG	Phase 1	Ch7 (Left MG)	Peak Amplitude EMG7	UPDRS Part III	101	-0.028	0.785
sEMG	Phase 1	Ch7 (Left MG)	Peak Amplitude EMG7	UPDRS total	101	0.057	0.573
sEMG	Phase 1	Ch8 (Right MG)	Peak Amplitude EMG8	H&Y stage	101	0.065	0.517
sEMG	Phase 1	Ch8 (Right MG)	Peak Amplitude EMG8	UPDRS Part III	101	-0.136	0.174
sEMG	Phase 1	Ch8 (Right MG)	Peak Amplitude EMG8	UPDRS total	101	-0.031	0.762
sEMG	Phase 1	Left (Left RF–BF)	Co-contraction IndexL	H&Y stage	101	-0.216	0.030
sEMG	Phase 1	Left (Left RF–BF)	Co-contraction IndexL	UPDRS Part III	101	-0.178	0.076

sEMG	Phase 1	Left (Left RF–BF)	Co-contraction IndexL	UPDRS total	101	-0.175	0.079
sEMG	Phase 1	Right (Right RF–BF)	Co-contraction IndexR	H&Y stage	101	-0.018	0.856
sEMG	Phase 1	Right (Right RF–BF)	Co-contraction IndexR	UPDRS Part III	101	-0.095	0.345
sEMG	Phase 1	Right (Right RF–BF)	Co-contraction IndexR	UPDRS total	101	-0.047	0.641
sEMG	Phase 1	—	Median Frequency	H&Y stage	101	0.115	0.251
sEMG	Phase 1	—	Median Frequency	UPDRS Part III	101	0.073	0.470
sEMG	Phase 1	—	Median Frequency	UPDRS total	101	0.018	0.858
sEMG	Phase 1	—	Mean Frequency	H&Y stage	101	0.135	0.179
sEMG	Phase 1	—	Mean Frequency	UPDRS Part III	101	0.076	0.449
sEMG	Phase 1	—	Mean Frequency	UPDRS total	101	0.032	0.750
sEMG	Phase 1	—	Frequency Band Power	H&Y stage	101	-0.025	0.802
sEMG	Phase 1	—	Frequency Band Power	UPDRS Part III	101	-0.010	0.922
sEMG	Phase 1	—	Frequency Band Power	UPDRS total	101	-0.100	0.322
sEMG	Phase 1	—	Entropy	H&Y stage	101	0.039	0.700
sEMG	Phase 1	—	Entropy	UPDRS Part III	101	-0.071	0.480
sEMG	Phase 1	—	Entropy	UPDRS total	101	-0.151	0.131
sEMG	Phase 1	—	AP SD	H&Y stage	101	0.003	0.973
sEMG	Phase 1	—	AP SD	UPDRS Part III	101	-0.034	0.739
sEMG	Phase 1	—	AP SD	UPDRS total	101	-0.158	0.115
sEMG	Phase 1	—	ML SD	H&Y stage	101	-0.029	0.774
sEMG	Phase 1	—	ML SD	UPDRS Part III	101	-0.070	0.488
sEMG	Phase 1	—	ML SD	UPDRS total	101	-0.167	0.095
sEMG	Phase 2	Ch1 (Left RF)	EMG1 RMS	H&Y stage	101	-0.003	0.977
sEMG	Phase 2	Ch1 (Left RF)	EMG1 RMS	UPDRS Part III	101	-0.043	0.668
sEMG	Phase 2	Ch1 (Left RF)	EMG1 RMS	UPDRS total	101	-0.146	0.146
sEMG	Phase 2	Ch2 (Right RF)	EMG2 RMS	H&Y stage	101	0.236	0.017
sEMG	Phase 2	Ch2 (Right RF)	EMG2 RMS	UPDRS Part III	101	0.180	0.071
sEMG	Phase 2	Ch2 (Right RF)	EMG2 RMS	UPDRS total	101	0.219	0.028
sEMG	Phase 2	Ch3 (Left TA)	EMG3 RMS	H&Y stage	101	-0.133	0.185
sEMG	Phase 2	Ch3 (Left TA)	EMG3 RMS	UPDRS Part III	101	-0.138	0.170
sEMG	Phase 2	Ch3 (Left TA)	EMG3 RMS	UPDRS total	101	-0.223	0.025
sEMG	Phase 2	Ch4 (Right TA)	EMG4 RMS	H&Y stage	101	0.211	0.034
sEMG	Phase 2	Ch4 (Right TA)	EMG4 RMS	UPDRS Part III	101	0.157	0.116
sEMG	Phase 2	Ch4 (Right TA)	EMG4 RMS	UPDRS total	101	0.140	0.163
sEMG	Phase 2	Ch5 (Left BF)	EMG5 RMS	H&Y stage	101	0.071	0.483
sEMG	Phase 2	Ch5 (Left BF)	EMG5 RMS	UPDRS Part III	101	0.107	0.289
sEMG	Phase 2	Ch5 (Left BF)	EMG5 RMS	UPDRS total	101	0.046	0.649
sEMG	Phase 2	Ch6 (Right BF)	EMG6 RMS	H&Y stage	101	0.048	0.635
sEMG	Phase 2	Ch6 (Right BF)	EMG6 RMS	UPDRS Part III	101	0.083	0.411
sEMG	Phase 2	Ch6 (Right BF)	EMG6 RMS	UPDRS total	101	0.090	0.371
sEMG	Phase 2	Ch7 (Left MG)	EMG7 RMS	H&Y stage	101	-0.026	0.798
sEMG	Phase 2	Ch7 (Left MG)	EMG7 RMS	UPDRS Part III	101	-0.004	0.967
sEMG	Phase 2	Ch7 (Left MG)	EMG7 RMS	UPDRS total	101	0.032	0.748
sEMG	Phase 2	Ch8 (Right MG)	EMG8 RMS	H&Y stage	101	0.074	0.461
sEMG	Phase 2	Ch8 (Right MG)	EMG8 RMS	UPDRS Part III	101	-0.082	0.413
sEMG	Phase 2	Ch8 (Right MG)	EMG8 RMS	UPDRS total	101	0.014	0.890
sEMG	Phase 2	Ch1 (Left RF)	Integrated EMG1	H&Y stage	101	0.049	0.625
sEMG	Phase 2	Ch1 (Left RF)	Integrated EMG1	UPDRS Part III	101	-0.067	0.503
sEMG	Phase 2	Ch1 (Left RF)	Integrated EMG1	UPDRS total	101	-0.144	0.150
sEMG	Phase 2	Ch2 (Right RF)	Integrated EMG2	H&Y stage	101	0.269	0.007
sEMG	Phase 2	Ch2 (Right RF)	Integrated EMG2	UPDRS Part III	101	0.144	0.151
sEMG	Phase 2	Ch2 (Right RF)	Integrated EMG2	UPDRS total	101	0.186	0.062
sEMG	Phase 2	Ch3 (Left TA)	Integrated EMG3	H&Y stage	101	-0.101	0.315
sEMG	Phase 2	Ch3 (Left TA)	Integrated EMG3	UPDRS Part III	101	-0.156	0.119
sEMG	Phase 2	Ch3 (Left TA)	Integrated EMG3	UPDRS total	101	-0.223	0.025
sEMG	Phase 2	Ch4 (Right TA)	Integrated EMG4	H&Y stage	101	0.228	0.022
sEMG	Phase 2	Ch4 (Right TA)	Integrated EMG4	UPDRS Part III	101	0.100	0.321
sEMG	Phase 2	Ch4 (Right TA)	Integrated EMG4	UPDRS total	101	0.106	0.291
sEMG	Phase 2	Ch5 (Left BF)	Integrated EMG5	H&Y stage	101	0.114	0.256
sEMG	Phase 2	Ch5 (Left BF)	Integrated EMG5	UPDRS Part III	101	0.050	0.617
sEMG	Phase 2	Ch5 (Left BF)	Integrated EMG5	UPDRS total	101	0.034	0.738
sEMG	Phase 2	Ch6 (Right BF)	Integrated EMG6	H&Y stage	101	0.080	0.428
sEMG	Phase 2	Ch6 (Right BF)	Integrated EMG6	UPDRS Part III	101	0.037	0.711
sEMG	Phase 2	Ch6 (Right BF)	Integrated EMG6	UPDRS total	101	0.073	0.468
sEMG	Phase 2	Ch7 (Left MG)	Integrated EMG7	H&Y stage	101	0.010	0.924
sEMG	Phase 2	Ch7 (Left MG)	Integrated EMG7	UPDRS Part III	101	0.008	0.934
sEMG	Phase 2	Ch7 (Left MG)	Integrated EMG7	UPDRS total	101	0.049	0.626
sEMG	Phase 2	Ch8 (Right MG)	Integrated EMG8	H&Y stage	101	0.089	0.379
sEMG	Phase 2	Ch8 (Right MG)	Integrated EMG8	UPDRS Part III	101	-0.092	0.360
sEMG	Phase 2	Ch8 (Right MG)	Integrated EMG8	UPDRS total	101	0.008	0.934
sEMG	Phase 2	Ch1 (Left RF)	Peak Amplitude EMG1	H&Y stage	101	0.010	0.920
sEMG	Phase 2	Ch1 (Left RF)	Peak Amplitude EMG1	UPDRS Part III	101	-0.042	0.679
sEMG	Phase 2	Ch1 (Left RF)	Peak Amplitude EMG1	UPDRS total	101	-0.138	0.167
sEMG	Phase 2	Ch2 (Right RF)	Peak Amplitude EMG2	H&Y stage	101	0.256	0.010
sEMG	Phase 2	Ch2 (Right RF)	Peak Amplitude EMG2	UPDRS Part III	101	0.188	0.059
sEMG	Phase 2	Ch2 (Right RF)	Peak Amplitude EMG2	UPDRS total	101	0.239	0.016
sEMG	Phase 2	Ch3 (Left TA)	Peak Amplitude EMG3	H&Y stage	101	-0.111	0.270
sEMG	Phase 2	Ch3 (Left TA)	Peak Amplitude EMG3	UPDRS Part III	101	-0.109	0.280
sEMG	Phase 2	Ch3 (Left TA)	Peak Amplitude EMG3	UPDRS total	101	-0.190	0.057
sEMG	Phase 2	Ch4 (Right TA)	Peak Amplitude EMG4	H&Y stage	101	0.226	0.023

sEMG	Phase 2	Ch4 (Right TA)	Peak Amplitude EMG4	UPDRS Part III	101	0.162	0.106
sEMG	Phase 2	Ch4 (Right TA)	Peak Amplitude EMG4	UPDRS total	101	0.163	0.104
sEMG	Phase 2	Ch5 (Left BF)	Peak Amplitude EMG5	H&Y stage	101	0.108	0.282
sEMG	Phase 2	Ch5 (Left BF)	Peak Amplitude EMG5	UPDRS Part III	101	0.115	0.253
sEMG	Phase 2	Ch5 (Left BF)	Peak Amplitude EMG5	UPDRS total	101	0.059	0.559
sEMG	Phase 2	Ch6 (Right BF)	Peak Amplitude EMG6	H&Y stage	101	0.062	0.537
sEMG	Phase 2	Ch6 (Right BF)	Peak Amplitude EMG6	UPDRS Part III	101	0.083	0.409
sEMG	Phase 2	Ch6 (Right BF)	Peak Amplitude EMG6	UPDRS total	101	0.103	0.305
sEMG	Phase 2	Ch7 (Left MG)	Peak Amplitude EMG7	H&Y stage	101	0.004	0.971
sEMG	Phase 2	Ch7 (Left MG)	Peak Amplitude EMG7	UPDRS Part III	101	0.020	0.842
sEMG	Phase 2	Ch7 (Left MG)	Peak Amplitude EMG7	UPDRS total	101	0.060	0.552
sEMG	Phase 2	Ch8 (Right MG)	Peak Amplitude EMG8	H&Y stage	101	0.091	0.365
sEMG	Phase 2	Ch8 (Right MG)	Peak Amplitude EMG8	UPDRS Part III	101	-0.074	0.463
sEMG	Phase 2	Ch8 (Right MG)	Peak Amplitude EMG8	UPDRS total	101	0.041	0.682
sEMG	Phase 2	Left (Left RF–BF)	Co-contraction IndexL_1	H&Y stage	101	-0.095	0.345
sEMG	Phase 2	Left (Left RF–BF)	Co-contraction IndexL_1	UPDRS Part III	101	-0.100	0.322
sEMG	Phase 2	Left (Left RF–BF)	Co-contraction IndexL_1	UPDRS total	101	-0.040	0.693
sEMG	Phase 2	Right (Right RF–BF)	Co-contraction IndexR_1	H&Y stage	101	-0.045	0.657
sEMG	Phase 2	Right (Right RF–BF)	Co-contraction IndexR_1	UPDRS Part III	101	-0.024	0.810
sEMG	Phase 2	Right (Right RF–BF)	Co-contraction IndexR_1	UPDRS total	101	0.026	0.798
sEMG	Phase 2	Left (Left RF–BF)	Co-contraction IndexL_2	H&Y stage	101	-0.027	0.788
sEMG	Phase 2	Left (Left RF–BF)	Co-contraction IndexL_2	UPDRS Part III	101	-0.021	0.834
sEMG	Phase 2	Left (Left RF–BF)	Co-contraction IndexL_2	UPDRS total	101	0.045	0.656
sEMG	Phase 2	Right (Right RF–BF)	Co-contraction IndexR_2	H&Y stage	101	0.038	0.708
sEMG	Phase 2	Right (Right RF–BF)	Co-contraction IndexR_2	UPDRS Part III	101	0.000	0.998
sEMG	Phase 2	Right (Right RF–BF)	Co-contraction IndexR_2	UPDRS total	101	0.050	0.622
sEMG	Phase 2	—	Median Frequency	H&Y stage	101	-0.082	0.416
sEMG	Phase 2	—	Median Frequency	UPDRS Part III	101	-0.177	0.077
sEMG	Phase 2	—	Median Frequency	UPDRS total	101	-0.160	0.109
sEMG	Phase 2	—	Mean Frequency	H&Y stage	101	-0.093	0.356
sEMG	Phase 2	—	Mean Frequency	UPDRS Part III	101	-0.158	0.116
sEMG	Phase 2	—	Mean Frequency	UPDRS total	101	-0.157	0.118
sEMG	Phase 2	—	Frequency Band Power	H&Y stage	101	-0.015	0.884
sEMG	Phase 2	—	Frequency Band Power	UPDRS Part III	101	-0.014	0.892
sEMG	Phase 2	—	Frequency Band Power	UPDRS total	101	-0.076	0.449
sEMG	Phase 2	—	Entropy	H&Y stage	101	0.067	0.504
sEMG	Phase 2	—	Entropy	UPDRS Part III	101	-0.043	0.668
sEMG	Phase 2	—	Entropy	UPDRS total	101	-0.112	0.263
sEMG	Phase 2	—	AP SD	H&Y stage	101	0.040	0.688
sEMG	Phase 2	—	AP SD	UPDRS Part III	101	-0.044	0.661
sEMG	Phase 2	—	AP SD	UPDRS total	101	-0.121	0.230
sEMG	Phase 2	—	ML SD	H&Y stage	101	0.210	0.035
sEMG	Phase 2	—	ML SD	UPDRS Part III	101	0.196	0.049
sEMG	Phase 2	—	ML SD	UPDRS total	101	0.218	0.029
Grad-CAM	Overall STW	Ch1 (Left RF)	MaxIdx	H&Y stage	101	0.024	0.815
Grad-CAM	Overall STW	Ch1 (Left RF)	MaxIdx	UPDRS Part III	101	0.013	0.894
Grad-CAM	Overall STW	Ch1 (Left RF)	MaxIdx	UPDRS total	101	0.073	0.470
Grad-CAM	Overall STW	Ch1 (Left RF)	CenterIdx	H&Y stage	101	0.026	0.796
Grad-CAM	Overall STW	Ch1 (Left RF)	CenterIdx	UPDRS Part III	101	-0.025	0.803
Grad-CAM	Overall STW	Ch1 (Left RF)	CenterIdx	UPDRS total	101	0.017	0.865
Grad-CAM	Overall STW	Ch1 (Left RF)	HalfEnergyIdx	H&Y stage	101	0.022	0.829
Grad-CAM	Overall STW	Ch1 (Left RF)	HalfEnergyIdx	UPDRS Part III	101	-0.028	0.779
Grad-CAM	Overall STW	Ch1 (Left RF)	HalfEnergyIdx	UPDRS total	101	0.007	0.947
Grad-CAM	Overall STW	Ch2 (Right RF)	MaxIdx	H&Y stage	101	0.052	0.605
Grad-CAM	Overall STW	Ch2 (Right RF)	MaxIdx	UPDRS Part III	101	-0.062	0.541
Grad-CAM	Overall STW	Ch2 (Right RF)	MaxIdx	UPDRS total	101	-0.081	0.422
Grad-CAM	Overall STW	Ch2 (Right RF)	CenterIdx	H&Y stage	101	-0.008	0.937
Grad-CAM	Overall STW	Ch2 (Right RF)	CenterIdx	UPDRS Part III	101	0.007	0.948
Grad-CAM	Overall STW	Ch2 (Right RF)	CenterIdx	UPDRS total	101	-0.140	0.163
Grad-CAM	Overall STW	Ch2 (Right RF)	HalfEnergyIdx	H&Y stage	101	-0.007	0.944
Grad-CAM	Overall STW	Ch2 (Right RF)	HalfEnergyIdx	UPDRS Part III	101	-0.006	0.955
Grad-CAM	Overall STW	Ch2 (Right RF)	HalfEnergyIdx	UPDRS total	101	-0.152	0.128
Grad-CAM	Overall STW	Ch3 (Left TA)	MaxIdx	H&Y stage	101	-0.158	0.114
Grad-CAM	Overall STW	Ch3 (Left TA)	MaxIdx	UPDRS Part III	101	-0.112	0.266
Grad-CAM	Overall STW	Ch3 (Left TA)	MaxIdx	UPDRS total	101	-0.127	0.207
Grad-CAM	Overall STW	Ch3 (Left TA)	CenterIdx	H&Y stage	101	-0.216	0.030
Grad-CAM	Overall STW	Ch3 (Left TA)	CenterIdx	UPDRS Part III	101	-0.131	0.193
Grad-CAM	Overall STW	Ch3 (Left TA)	CenterIdx	UPDRS total	101	-0.157	0.116
Grad-CAM	Overall STW	Ch3 (Left TA)	HalfEnergyIdx	H&Y stage	101	-0.184	0.066
Grad-CAM	Overall STW	Ch3 (Left TA)	HalfEnergyIdx	UPDRS Part III	101	-0.152	0.129
Grad-CAM	Overall STW	Ch3 (Left TA)	HalfEnergyIdx	UPDRS total	101	-0.179	0.073
Grad-CAM	Overall STW	Ch4 (Right TA)	MaxIdx	H&Y stage	101	-0.125	0.214
Grad-CAM	Overall STW	Ch4 (Right TA)	MaxIdx	UPDRS Part III	101	-0.144	0.151
Grad-CAM	Overall STW	Ch4 (Right TA)	MaxIdx	UPDRS total	101	-0.208	0.036
Grad-CAM	Overall STW	Ch4 (Right TA)	CenterIdx	H&Y stage	101	-0.055	0.583
Grad-CAM	Overall STW	Ch4 (Right TA)	CenterIdx	UPDRS Part III	101	-0.098	0.328
Grad-CAM	Overall STW	Ch4 (Right TA)	CenterIdx	UPDRS total	101	-0.171	0.087
Grad-CAM	Overall STW	Ch4 (Right TA)	HalfEnergyIdx	H&Y stage	101	-0.091	0.366
Grad-CAM	Overall STW	Ch4 (Right TA)	HalfEnergyIdx	UPDRS Part III	101	-0.118	0.238
Grad-CAM	Overall STW	Ch4 (Right TA)	HalfEnergyIdx	UPDRS total	101	-0.198	0.047

Grad-CAM	Overall STW	Ch5 (Left BF)	MaxIdx	H&Y stage	101	0.056	0.576
Grad-CAM	Overall STW	Ch5 (Left BF)	MaxIdx	UPDRS Part III	101	-0.020	0.843
Grad-CAM	Overall STW	Ch5 (Left BF)	MaxIdx	UPDRS total	101	-0.011	0.912
Grad-CAM	Overall STW	Ch5 (Left BF)	CenterIdx	H&Y stage	101	0.056	0.576
Grad-CAM	Overall STW	Ch5 (Left BF)	CenterIdx	UPDRS Part III	101	0.025	0.804
Grad-CAM	Overall STW	Ch5 (Left BF)	CenterIdx	UPDRS total	101	0.051	0.613
Grad-CAM	Overall STW	Ch5 (Left BF)	HalfEnergyIdx	H&Y stage	101	0.075	0.455
Grad-CAM	Overall STW	Ch5 (Left BF)	HalfEnergyIdx	UPDRS Part III	101	0.007	0.942
Grad-CAM	Overall STW	Ch5 (Left BF)	HalfEnergyIdx	UPDRS total	101	0.034	0.733
Grad-CAM	Overall STW	Ch6 (Right BF)	MaxIdx	H&Y stage	101	-0.043	0.668
Grad-CAM	Overall STW	Ch6 (Right BF)	MaxIdx	UPDRS Part III	101	-0.142	0.156
Grad-CAM	Overall STW	Ch6 (Right BF)	MaxIdx	UPDRS total	101	-0.142	0.158
Grad-CAM	Overall STW	Ch6 (Right BF)	CenterIdx	H&Y stage	101	-0.028	0.782
Grad-CAM	Overall STW	Ch6 (Right BF)	CenterIdx	UPDRS Part III	101	-0.096	0.339
Grad-CAM	Overall STW	Ch6 (Right BF)	CenterIdx	UPDRS total	101	-0.058	0.567
Grad-CAM	Overall STW	Ch6 (Right BF)	HalfEnergyIdx	H&Y stage	101	-0.050	0.618
Grad-CAM	Overall STW	Ch6 (Right BF)	HalfEnergyIdx	UPDRS Part III	101	-0.117	0.243
Grad-CAM	Overall STW	Ch6 (Right BF)	HalfEnergyIdx	UPDRS total	101	-0.079	0.434
Grad-CAM	Overall STW	Ch7 (Left MG)	MaxIdx	H&Y stage	101	-0.054	0.590
Grad-CAM	Overall STW	Ch7 (Left MG)	MaxIdx	UPDRS Part III	101	0.081	0.419
Grad-CAM	Overall STW	Ch7 (Left MG)	MaxIdx	UPDRS total	101	0.062	0.541
Grad-CAM	Overall STW	Ch7 (Left MG)	CenterIdx	H&Y stage	101	0.003	0.977
Grad-CAM	Overall STW	Ch7 (Left MG)	CenterIdx	UPDRS Part III	101	0.087	0.389
Grad-CAM	Overall STW	Ch7 (Left MG)	CenterIdx	UPDRS total	101	0.037	0.713
Grad-CAM	Overall STW	Ch7 (Left MG)	HalfEnergyIdx	H&Y stage	101	-0.001	0.990
Grad-CAM	Overall STW	Ch7 (Left MG)	HalfEnergyIdx	UPDRS Part III	101	0.087	0.386
Grad-CAM	Overall STW	Ch7 (Left MG)	HalfEnergyIdx	UPDRS total	101	0.040	0.692
Grad-CAM	Overall STW	Ch8 (Right MG)	MaxIdx	H&Y stage	101	-0.084	0.402
Grad-CAM	Overall STW	Ch8 (Right MG)	MaxIdx	UPDRS Part III	101	-0.042	0.673
Grad-CAM	Overall STW	Ch8 (Right MG)	MaxIdx	UPDRS total	101	-0.046	0.650
Grad-CAM	Overall STW	Ch8 (Right MG)	CenterIdx	H&Y stage	101	-0.043	0.672
Grad-CAM	Overall STW	Ch8 (Right MG)	CenterIdx	UPDRS Part III	101	0.018	0.859
Grad-CAM	Overall STW	Ch8 (Right MG)	CenterIdx	UPDRS total	101	-0.008	0.939
Grad-CAM	Overall STW	Ch8 (Right MG)	HalfEnergyIdx	H&Y stage	101	-0.047	0.642
Grad-CAM	Overall STW	Ch8 (Right MG)	HalfEnergyIdx	UPDRS Part III	101	0.018	0.858
Grad-CAM	Overall STW	Ch8 (Right MG)	HalfEnergyIdx	UPDRS total	101	-0.015	0.878
Grad-CAM	Phase 1	Ch1 (Left RF)	MaxIdx	H&Y stage	101	-0.029	0.776
Grad-CAM	Phase 1	Ch1 (Left RF)	MaxIdx	UPDRS Part III	101	0.014	0.892
Grad-CAM	Phase 1	Ch1 (Left RF)	MaxIdx	UPDRS total	101	-0.016	0.871
Grad-CAM	Phase 1	Ch1 (Left RF)	CenterIdx	H&Y stage	101	0.000	0.997
Grad-CAM	Phase 1	Ch1 (Left RF)	CenterIdx	UPDRS Part III	101	0.077	0.445
Grad-CAM	Phase 1	Ch1 (Left RF)	CenterIdx	UPDRS total	101	-0.026	0.797
Grad-CAM	Phase 1	Ch1 (Left RF)	HalfEnergyIdx	H&Y stage	101	0.049	0.625
Grad-CAM	Phase 1	Ch1 (Left RF)	HalfEnergyIdx	UPDRS Part III	101	0.154	0.125
Grad-CAM	Phase 1	Ch1 (Left RF)	HalfEnergyIdx	UPDRS total	101	0.061	0.548
Grad-CAM	Phase 1	Ch2 (Right RF)	MaxIdx	H&Y stage	101	-0.055	0.588
Grad-CAM	Phase 1	Ch2 (Right RF)	MaxIdx	UPDRS Part III	101	-0.053	0.598
Grad-CAM	Phase 1	Ch2 (Right RF)	MaxIdx	UPDRS total	101	-0.078	0.441
Grad-CAM	Phase 1	Ch2 (Right RF)	CenterIdx	H&Y stage	101	-0.087	0.390
Grad-CAM	Phase 1	Ch2 (Right RF)	CenterIdx	UPDRS Part III	101	-0.099	0.324
Grad-CAM	Phase 1	Ch2 (Right RF)	CenterIdx	UPDRS total	101	-0.167	0.095
Grad-CAM	Phase 1	Ch2 (Right RF)	HalfEnergyIdx	H&Y stage	101	-0.034	0.735
Grad-CAM	Phase 1	Ch2 (Right RF)	HalfEnergyIdx	UPDRS Part III	101	-0.031	0.760
Grad-CAM	Phase 1	Ch2 (Right RF)	HalfEnergyIdx	UPDRS total	101	-0.120	0.231
Grad-CAM	Phase 1	Ch3 (Left TA)	MaxIdx	H&Y stage	101	-0.030	0.766
Grad-CAM	Phase 1	Ch3 (Left TA)	MaxIdx	UPDRS Part III	101	0.142	0.157
Grad-CAM	Phase 1	Ch3 (Left TA)	MaxIdx	UPDRS total	101	0.049	0.628
Grad-CAM	Phase 1	Ch3 (Left TA)	CenterIdx	H&Y stage	101	-0.020	0.843
Grad-CAM	Phase 1	Ch3 (Left TA)	CenterIdx	UPDRS Part III	101	0.161	0.109
Grad-CAM	Phase 1	Ch3 (Left TA)	CenterIdx	UPDRS total	101	0.064	0.523
Grad-CAM	Phase 1	Ch3 (Left TA)	HalfEnergyIdx	H&Y stage	101	-0.137	0.172
Grad-CAM	Phase 1	Ch3 (Left TA)	HalfEnergyIdx	UPDRS Part III	101	0.003	0.979
Grad-CAM	Phase 1	Ch3 (Left TA)	HalfEnergyIdx	UPDRS total	101	-0.109	0.279
Grad-CAM	Phase 1	Ch4 (Right TA)	MaxIdx	H&Y stage	101	-0.071	0.479
Grad-CAM	Phase 1	Ch4 (Right TA)	MaxIdx	UPDRS Part III	101	-0.051	0.611
Grad-CAM	Phase 1	Ch4 (Right TA)	MaxIdx	UPDRS total	101	-0.041	0.683
Grad-CAM	Phase 1	Ch4 (Right TA)	CenterIdx	H&Y stage	101	-0.032	0.752
Grad-CAM	Phase 1	Ch4 (Right TA)	CenterIdx	UPDRS Part III	101	-0.015	0.884
Grad-CAM	Phase 1	Ch4 (Right TA)	CenterIdx	UPDRS total	101	-0.053	0.601
Grad-CAM	Phase 1	Ch4 (Right TA)	HalfEnergyIdx	H&Y stage	101	-0.006	0.950
Grad-CAM	Phase 1	Ch4 (Right TA)	HalfEnergyIdx	UPDRS Part III	101	-0.027	0.792
Grad-CAM	Phase 1	Ch4 (Right TA)	HalfEnergyIdx	UPDRS total	101	-0.067	0.506
Grad-CAM	Phase 1	Ch5 (Left BF)	MaxIdx	H&Y stage	101	-0.132	0.189
Grad-CAM	Phase 1	Ch5 (Left BF)	MaxIdx	UPDRS Part III	101	-0.111	0.270
Grad-CAM	Phase 1	Ch5 (Left BF)	MaxIdx	UPDRS total	101	-0.082	0.412
Grad-CAM	Phase 1	Ch5 (Left BF)	CenterIdx	H&Y stage	101	-0.081	0.422
Grad-CAM	Phase 1	Ch5 (Left BF)	CenterIdx	UPDRS Part III	101	0.007	0.946
Grad-CAM	Phase 1	Ch5 (Left BF)	CenterIdx	UPDRS total	101	-0.058	0.566
Grad-CAM	Phase 1	Ch5 (Left BF)	HalfEnergyIdx	H&Y stage	101	-0.025	0.801
Grad-CAM	Phase 1	Ch5 (Left BF)	HalfEnergyIdx	UPDRS Part III	101	0.050	0.618

Grad-CAM	Phase 1	Ch5 (Left BF)	HalfEnergyIdx	UPDRS total	101	-0.010	0.920
Grad-CAM	Phase 1	Ch6 (Right BF)	MaxIdx	H&Y stage	101	0.012	0.902
Grad-CAM	Phase 1	Ch6 (Right BF)	MaxIdx	UPDRS Part III	101	0.110	0.272
Grad-CAM	Phase 1	Ch6 (Right BF)	MaxIdx	UPDRS total	101	0.042	0.679
Grad-CAM	Phase 1	Ch6 (Right BF)	CenterIdx	H&Y stage	101	-0.107	0.285
Grad-CAM	Phase 1	Ch6 (Right BF)	CenterIdx	UPDRS Part III	101	-0.003	0.976
Grad-CAM	Phase 1	Ch6 (Right BF)	CenterIdx	UPDRS total	101	-0.092	0.358
Grad-CAM	Phase 1	Ch6 (Right BF)	HalfEnergyIdx	H&Y stage	101	-0.089	0.377
Grad-CAM	Phase 1	Ch6 (Right BF)	HalfEnergyIdx	UPDRS Part III	101	-0.016	0.875
Grad-CAM	Phase 1	Ch6 (Right BF)	HalfEnergyIdx	UPDRS total	101	-0.073	0.466
Grad-CAM	Phase 1	Ch7 (Left MG)	MaxIdx	H&Y stage	101	-0.092	0.359
Grad-CAM	Phase 1	Ch7 (Left MG)	MaxIdx	UPDRS Part III	101	0.017	0.865
Grad-CAM	Phase 1	Ch7 (Left MG)	MaxIdx	UPDRS total	101	-0.017	0.868
Grad-CAM	Phase 1	Ch7 (Left MG)	CenterIdx	H&Y stage	101	0.010	0.917
Grad-CAM	Phase 1	Ch7 (Left MG)	CenterIdx	UPDRS Part III	101	0.006	0.951
Grad-CAM	Phase 1	Ch7 (Left MG)	CenterIdx	UPDRS total	101	0.050	0.623
Grad-CAM	Phase 1	Ch7 (Left MG)	HalfEnergyIdx	H&Y stage	101	-0.015	0.883
Grad-CAM	Phase 1	Ch7 (Left MG)	HalfEnergyIdx	UPDRS Part III	101	-0.007	0.942
Grad-CAM	Phase 1	Ch7 (Left MG)	HalfEnergyIdx	UPDRS total	101	0.029	0.776
Grad-CAM	Phase 1	Ch8 (Right MG)	MaxIdx	H&Y stage	101	0.045	0.657
Grad-CAM	Phase 1	Ch8 (Right MG)	MaxIdx	UPDRS Part III	101	0.025	0.801
Grad-CAM	Phase 1	Ch8 (Right MG)	MaxIdx	UPDRS total	101	0.064	0.522
Grad-CAM	Phase 1	Ch8 (Right MG)	CenterIdx	H&Y stage	101	-0.076	0.453
Grad-CAM	Phase 1	Ch8 (Right MG)	CenterIdx	UPDRS Part III	101	-0.167	0.095
Grad-CAM	Phase 1	Ch8 (Right MG)	CenterIdx	UPDRS total	101	-0.120	0.233
Grad-CAM	Phase 1	Ch8 (Right MG)	HalfEnergyIdx	H&Y stage	101	-0.059	0.555
Grad-CAM	Phase 1	Ch8 (Right MG)	HalfEnergyIdx	UPDRS Part III	101	-0.146	0.146
Grad-CAM	Phase 1	Ch8 (Right MG)	HalfEnergyIdx	UPDRS total	101	-0.107	0.287
Grad-CAM	Phase 2	Ch1 (Left RF)	MaxIdx	H&Y stage	101	0.006	0.955
Grad-CAM	Phase 2	Ch1 (Left RF)	MaxIdx	UPDRS Part III	101	-0.242	0.015
Grad-CAM	Phase 2	Ch1 (Left RF)	MaxIdx	UPDRS total	101	-0.200	0.045
Grad-CAM	Phase 2	Ch1 (Left RF)	CenterIdx	H&Y stage	101	0.014	0.892
Grad-CAM	Phase 2	Ch1 (Left RF)	CenterIdx	UPDRS Part III	101	-0.009	0.928
Grad-CAM	Phase 2	Ch1 (Left RF)	CenterIdx	UPDRS total	101	-0.007	0.944
Grad-CAM	Phase 2	Ch1 (Left RF)	HalfEnergyIdx	H&Y stage	101	-0.009	0.933
Grad-CAM	Phase 2	Ch1 (Left RF)	HalfEnergyIdx	UPDRS Part III	101	0.016	0.873
Grad-CAM	Phase 2	Ch1 (Left RF)	HalfEnergyIdx	UPDRS total	101	0.015	0.881
Grad-CAM	Phase 2	Ch2 (Right RF)	MaxIdx	H&Y stage	101	-0.004	0.968
Grad-CAM	Phase 2	Ch2 (Right RF)	MaxIdx	UPDRS Part III	101	0.024	0.810
Grad-CAM	Phase 2	Ch2 (Right RF)	MaxIdx	UPDRS total	101	0.053	0.602
Grad-CAM	Phase 2	Ch2 (Right RF)	CenterIdx	H&Y stage	101	-0.074	0.464
Grad-CAM	Phase 2	Ch2 (Right RF)	CenterIdx	UPDRS Part III	101	0.027	0.792
Grad-CAM	Phase 2	Ch2 (Right RF)	CenterIdx	UPDRS total	101	0.071	0.483
Grad-CAM	Phase 2	Ch2 (Right RF)	HalfEnergyIdx	H&Y stage	101	0.021	0.832
Grad-CAM	Phase 2	Ch2 (Right RF)	HalfEnergyIdx	UPDRS Part III	101	0.121	0.228
Grad-CAM	Phase 2	Ch2 (Right RF)	HalfEnergyIdx	UPDRS total	101	0.127	0.205
Grad-CAM	Phase 2	Ch3 (Left TA)	MaxIdx	H&Y stage	101	0.170	0.089
Grad-CAM	Phase 2	Ch3 (Left TA)	MaxIdx	UPDRS Part III	101	0.168	0.094
Grad-CAM	Phase 2	Ch3 (Left TA)	MaxIdx	UPDRS total	101	0.185	0.064
Grad-CAM	Phase 2	Ch3 (Left TA)	CenterIdx	H&Y stage	101	0.035	0.727
Grad-CAM	Phase 2	Ch3 (Left TA)	CenterIdx	UPDRS Part III	101	0.101	0.313
Grad-CAM	Phase 2	Ch3 (Left TA)	CenterIdx	UPDRS total	101	0.056	0.577
Grad-CAM	Phase 2	Ch3 (Left TA)	HalfEnergyIdx	H&Y stage	101	0.037	0.716
Grad-CAM	Phase 2	Ch3 (Left TA)	HalfEnergyIdx	UPDRS Part III	101	0.097	0.336
Grad-CAM	Phase 2	Ch3 (Left TA)	HalfEnergyIdx	UPDRS total	101	0.038	0.705
Grad-CAM	Phase 2	Ch4 (Right TA)	MaxIdx	H&Y stage	101	-0.186	0.062
Grad-CAM	Phase 2	Ch4 (Right TA)	MaxIdx	UPDRS Part III	101	-0.058	0.563
Grad-CAM	Phase 2	Ch4 (Right TA)	MaxIdx	UPDRS total	101	-0.101	0.315
Grad-CAM	Phase 2	Ch4 (Right TA)	CenterIdx	H&Y stage	101	-0.111	0.267
Grad-CAM	Phase 2	Ch4 (Right TA)	CenterIdx	UPDRS Part III	101	0.015	0.884
Grad-CAM	Phase 2	Ch4 (Right TA)	CenterIdx	UPDRS total	101	-0.016	0.875
Grad-CAM	Phase 2	Ch4 (Right TA)	HalfEnergyIdx	H&Y stage	101	-0.105	0.298
Grad-CAM	Phase 2	Ch4 (Right TA)	HalfEnergyIdx	UPDRS Part III	101	0.069	0.491
Grad-CAM	Phase 2	Ch4 (Right TA)	HalfEnergyIdx	UPDRS total	101	0.033	0.743
Grad-CAM	Phase 2	Ch5 (Left BF)	MaxIdx	H&Y stage	101	0.095	0.344
Grad-CAM	Phase 2	Ch5 (Left BF)	MaxIdx	UPDRS Part III	101	0.019	0.853
Grad-CAM	Phase 2	Ch5 (Left BF)	MaxIdx	UPDRS total	101	0.015	0.885
Grad-CAM	Phase 2	Ch5 (Left BF)	CenterIdx	H&Y stage	101	0.136	0.175
Grad-CAM	Phase 2	Ch5 (Left BF)	CenterIdx	UPDRS Part III	101	-0.006	0.953
Grad-CAM	Phase 2	Ch5 (Left BF)	CenterIdx	UPDRS total	101	-0.024	0.809
Grad-CAM	Phase 2	Ch5 (Left BF)	HalfEnergyIdx	H&Y stage	101	0.128	0.201
Grad-CAM	Phase 2	Ch5 (Left BF)	HalfEnergyIdx	UPDRS Part III	101	0.008	0.940
Grad-CAM	Phase 2	Ch5 (Left BF)	HalfEnergyIdx	UPDRS total	101	-0.013	0.899
Grad-CAM	Phase 2	Ch6 (Right BF)	MaxIdx	H&Y stage	101	-0.132	0.188
Grad-CAM	Phase 2	Ch6 (Right BF)	MaxIdx	UPDRS Part III	101	-0.050	0.618
Grad-CAM	Phase 2	Ch6 (Right BF)	MaxIdx	UPDRS total	101	-0.089	0.374
Grad-CAM	Phase 2	Ch6 (Right BF)	CenterIdx	H&Y stage	101	-0.092	0.362
Grad-CAM	Phase 2	Ch6 (Right BF)	CenterIdx	UPDRS Part III	101	-0.021	0.835
Grad-CAM	Phase 2	Ch6 (Right BF)	CenterIdx	UPDRS total	101	-0.061	0.546
Grad-CAM	Phase 2	Ch6 (Right BF)	HalfEnergyIdx	H&Y stage	101	-0.071	0.482

Grad-CAM	Phase 2	Ch6 (Right BF)	HalfEnergyIdx	UPDRS Part III	101	-0.011	0.912
Grad-CAM	Phase 2	Ch6 (Right BF)	HalfEnergyIdx	UPDRS total	101	-0.065	0.517
Grad-CAM	Phase 2	Ch7 (Left MG)	MaxIdx	H&Y stage	101	0.037	0.714
Grad-CAM	Phase 2	Ch7 (Left MG)	MaxIdx	UPDRS Part III	101	0.130	0.194
Grad-CAM	Phase 2	Ch7 (Left MG)	MaxIdx	UPDRS total	101	0.059	0.560
Grad-CAM	Phase 2	Ch7 (Left MG)	CenterIdx	H&Y stage	101	0.175	0.080
Grad-CAM	Phase 2	Ch7 (Left MG)	CenterIdx	UPDRS Part III	101	0.225	0.024
Grad-CAM	Phase 2	Ch7 (Left MG)	CenterIdx	UPDRS total	101	0.208	0.037
Grad-CAM	Phase 2	Ch7 (Left MG)	HalfEnergyIdx	H&Y stage	101	0.142	0.158
Grad-CAM	Phase 2	Ch7 (Left MG)	HalfEnergyIdx	UPDRS Part III	101	0.227	0.023
Grad-CAM	Phase 2	Ch7 (Left MG)	HalfEnergyIdx	UPDRS total	101	0.200	0.045
Grad-CAM	Phase 2	Ch8 (Right MG)	MaxIdx	H&Y stage	101	0.061	0.543
Grad-CAM	Phase 2	Ch8 (Right MG)	MaxIdx	UPDRS Part III	101	0.138	0.169
Grad-CAM	Phase 2	Ch8 (Right MG)	MaxIdx	UPDRS total	101	0.110	0.275
Grad-CAM	Phase 2	Ch8 (Right MG)	CenterIdx	H&Y stage	101	-0.040	0.691
Grad-CAM	Phase 2	Ch8 (Right MG)	CenterIdx	UPDRS Part III	101	0.076	0.452
Grad-CAM	Phase 2	Ch8 (Right MG)	CenterIdx	UPDRS total	101	0.039	0.702
Grad-CAM	Phase 2	Ch8 (Right MG)	HalfEnergyIdx	H&Y stage	101	-0.001	0.996
Grad-CAM	Phase 2	Ch8 (Right MG)	HalfEnergyIdx	UPDRS Part III	101	0.118	0.239
Grad-CAM	Phase 2	Ch8 (Right MG)	HalfEnergyIdx	UPDRS total	101	0.032	0.747

Note. Spearman's rank correlation coefficients (ρ) are reported for all valid sEMG–clinical and Grad-CAM–clinical correlation results contained in the supplied analysis workbooks. Only clinical outcomes (H&Y stage, UPDRS Part III, and UPDRS total) and the 66 sEMG-derived variables / 72 Grad-CAM temporal indices were retained; identifier/laterality fields were excluded. P-values are unadjusted and should be interpreted as hypothesis-generating. $p<0.05$ denotes a nominal exploratory association and not statistical significance after multiple-comparison correction. RF, rectus femoris; TA, tibialis anterior; BF, biceps femoris; MG, medial gastrocnemius.
